# Phenotyping of post-fertilization sperm mitophagy determinants discovered in a mammalian gamete-based cell-free system

**DOI:** 10.1186/s40659-026-00713-x

**Published:** 2026-06-29

**Authors:** Alexis Jones, Natálie Zelenková, Erica Mantle, Chloe Gardner, Barbora Klusáčková, Dalen Zuidema, Miriam Sutovsky, Pavla Postlerová, Michal Zigo, Peter Sutovsky

**Affiliations:** 1 Division of Animal Sciences, University of Missouri, Columbia, MO 65211-5300 USA; 2 Department of Obstetrics, Gynecology and Women’s Health, University of Missouri, Columbia, MO 65211-5300 USA; 3 Department of Veterinary Sciences, Faculty of Agrobiology, Food, and Natural Resources, Czech University of Life Sciences , Prague, Czech Republic; 4 Laboratory of Reproductive Biology, Institute of Biotechnology of the Czech Academy of Sciences, BIOCEV, Vestec, Czech Republic

**Keywords:** Sperm, Fertilization, Zygote, Autophagy, Mitochondria, Inheritance

## Abstract

The targeted, substrate-specific degradation of paternal mitochondria inside the zygote, known as post-fertilization sperm mitophagy, is a crucial and evolutionarily conserved early embryonic event. It ensures the exclusive maternal inheritance of the mitochondrial genome. Post-fertilization sperm mitophagy was initially thought to only be achieved via the ubiquitin–proteasome system. Until pro-autophagic receptor proteins such as SQSTM1, GABARAP, as well as the proteasome-interacting ubiquitinated protein dislocase VCP, were identified as contributors to the degradation of the sperm mitochondria early after mammalian fertilization. This synergy of proteasomal and autophagic pathways ensures a timely degradation of sperm mitochondria shortly after fertilization. The discovery of these autophagic receptors lead researchers to believe there might be other autophagic receptors and determinants necessary for proper post-fertilization sperm mitophagy. Based on the established inventory of proteins from mass spectrometry trials of boar spermatozoa exposed to porcine oocyte extracts in an intra-specific porcine cell-free system (CFS), five candidate mitophagy determinants were further investigated in this study, namely LACTB, PRDX3, PSMA8, TOMM34, and FUNDC1. These proteins of interest were studied and validated by using in vitro fertilization (IVF) protocols, cell imaging of spermatids, spermatozoa, oocytes and zygotes, protein interactome analysis, and the porcine CFS. The proteins PSMA8 and TOMM34 behaved in accordance with our proteomic study predictions. The PSMA8 labeling increased after exposure to CFS; in agreement with the classification PSMA8 was given from the mass spectrometry findings. TOMM34 underwent a visible decrease in labeling after exposure to CFS, which also agreed with its proteomic classification; this labeling persisted in IVF zygotes. Except for LACTB, the examined proteins showed mutual interactions as well as interactions with previously identified sperm mitophagy factors in the STRING interactome analysis. Results from this study validate the novel porcine CFS as a valuable tool for the exploration of early fertilization events at a molecular level. Future phenotyping and functional studies using porcine CFS will advance the understanding of mitochondrial inheritance and zygotic development and potentially shed light on the origins of certain mitochondrial diseases arising from the failure of post-fertilization sperm mitophagy.

**Supplementary Information:**

The online version contains supplementary material available at https://doi.org/10.1186/s40659-026-00713-x.

## Introduction

Mitochondrial degradation, also known as mitophagy, is a process mediated by the combined action of the ubiquitin proteasome system (UPS) and autophagic pathways. The timely removal of the paternal, sperm-borne mitochondria is a crucial and evolutionarily conserved early fertilization event, which mediates maternal inheritance of mitochondrial DNA in mammals and assures their proper development. The pro-proteolytic tagging of sperm mitochondria by ubiquitination, a stable yet reversible post-translational modification, as well as the actions of the ubiquitin-dependent protein recycling by the 26S proteasome, has continued to be associated with the degradation of sperm mitochondria in early-stage mammalian embryos [1–4]. The autophagic pathway has been implicated in conjunction with the UPS in higher order mammals, including both pigs and rhesus monkeys, to degrade sperm-borne mitochondria early after fertilization [5]. Prior studies done in bovine and porcine zygotes suggest that sperm mitochondria are already tagged with ubiquitin during spermatogenesis, in the testis, and this ubiquitin tag, a self-imposed death sentence for sperm mitochondria, is not recognized until after sperm tail incorporation into the oocyte cytoplasm at fertilization [6]. Although several pro-autophagic proteins and their corresponding pathways have been implicated in the degradation of sperm mitochondria during early embryonic development, much remains unknown about this process. It is likely that there are other necessary autophagic proteins, cofactors, substrates, and determinant proteins involved in post-fertilization sperm mitophagy that have yet to be identified.

To fill in some of the gaps in knowledge surrounding post-fertilization sperm mitophagy, our laboratory devised a novel porcine cell-free system (CFS) which utilizes the cytosolic extract of mature metaphase II (MII) porcine oocytes as well as boar spermatozoa primed in order to mimic the stepwise accessory sperm structure processing during natural fertilization. This finely tuned CFS uses primed, partially demembranated spermatozoa as a bait for ooplasmic mitophagy proteins, allowing us to characterize gametic proteomic events responsible for the targeted degradation of paternal mitochondria inside the fertilized mammalian egg. In this unique porcine, semi-cell-free system, boar spermatozoa are exposed to a priming process in which their cellular membranes, both plasma and acrosomal, are removed by using lysolecithin. Following this, the boar spermatozoa are exposed to dithiothreitol (DTT) which removes disulfide bonds from stabilized accessory sperm structures, which are responsible for stabilizing both the mitochondrial sheath and the sperm head structures, including perinuclear theca and the nucleus [7]. By co-incubating porcine oocyte extract with these primed spermatozoa, proteomic interactions of early fertilization can be mimicked. This system has already been used and validated for the study of post-fertilization sperm mitophagy [8, 9]. However, it may have applications beyond this area of research, such as for the study of sperm chromatin structure before fertilization and its remodeling after fertilization, during the development of the paternal pronucleus. We have recently used this system in conjunction with quantitative mass spectrometry to develop an inventory of mitophagic proteins of interest. This mass spectrometry study [10] captured changes in protein abundances between primed and CFS-treated spermatozoa. The study was conducted in two separate sets of biological triplicates, with primed spermatozoa vs. spermatozoa exposed to the CFS for 4 h and with primed spermatozoa vs. spermatozoa exposed to the CFS for 24 h. An inventory of proteins, which showed trends of significant changes in abundance, was compiled from each of the trials. Furthermore, proteins were then classified based on their changes in abundance during the trials. The identified proteins were separated into three different classes. Class 1, proteins which were only detected on spermatozoa after co-incubation in the CFS and not detected on the primed spermatozoa prior to extract exposure; Class 2, proteins which were present on primed spermatozoa but underwent a significant increase in abundance after CFS exposure; and Class 3, proteins which were present on primed spermatozoa but were observed to decrease in abundance after CFS exposure. That study was conducted as an exploratory trial in order to derive a list of candidate mitophagy proteins, setting the stage for phenotypic validation of the select identified target proteins in porcine gametes and zygotes, which continues with the present study.

From the identified candidate mitophagy proteins, so far, we have been able to further investigate thirteen and publish phenotypic data on eight of them. The present report aims to validate five additional candidate pro-autophagic proteins, including lactamase beta (LACTB), peroxiredoxin 3 (PRDX3), proteasomal subunit alpha 8 (PSMA8), FUN14 domain containing 1 (FUNDC1), and translocase of outer mitochondrial membrane 34 (TOMM34). These five proteins are considered candidate mitophagy proteins of interest based on their known functions, as well as their identification in the porcine cell-free system via mass spectrometry. This investigation was an attempt to understand these proteins in more detail than what was inferred from the mass spectrometry data alone. To do this, we again utilized the porcine CFS, but this time in conjunction with immunocytochemistry and Western blotting, to characterize the localization pattern changes these sperm proteins underwent after exposure to oocyte extract. Additionally, we investigated these proteins in zygotes after IVF at different points of early development to characterize their localization patterns after *vitro* fertilization.

## Methodology

### Antibodies and probes

MitoTracker® Red CMXRos and DNA stain DAPI were purchased from Invitrogen (Molecular Probes, Invitrogen Carlsbad, CA, USA). Rabbit polyclonal anti-FUNDC1 (ab224722; diluted 1:50 for immunofluorescence) was purchased from Abcam (Abcam Limited, Cambridge, UK). Rabbit polyclonal anti-LACTB (PA5-57627; dil. 1:200 for immunofluorescence) was purchased from ThermoFisher (ThermoFisher Scientific, Waltham, MA, USA). Rabbit polyclonal anti-PRDX3 (PIPA591918; dil. 1:200 for immunofluorescence); rabbit polyclonal anti-PSMA8 (50-172-9159; dil. 1:100 for immunofluorescence); and rabbit polyclonal anti-TOMM34 (NBP220682; dil. 1:50 for immunofluorescence) were purchased from Fisher Scientific (Fisher Scientific Pittsburg, PA, USA). All primary antibodies were diluted 1:1,000 for Western blotting. Unless otherwise noted, all chemicals used in this study were purchased from Sigma Chemical Co. (St. Louis, MO, USA).

### Boar semen collection and processing

Domestic Large White cross boars were housed at the University of Missouri Animal Science Research Center. Fresh boar ejaculate was collected weekly, by the gloved hand technique then transferred into 15 mL conical tubes and centrifuged at 800 × g for 10 min to separate spermatozoa from seminal plasma. Sperm concentration was assessed by using a light microscope and a hemocytometer (ThermoFisher Scientific, Houston, TX, USA). Only semen collections with adequate sperm motility were used. Spermatozoa were diluted with *Preserv® Xtreme* boar extender (GenePro, WI) to a final concentration of 1 × 10^8^ spermatozoa/mL and stored at room temperature for up to 5 days. Sperm capacitation was performed in capacitation-promoting medium containing 2 mM Ca^2+^, 2 mM HCO_3_^−^, and 2% (w/v) BSA for four hours at 37 °C, as described in our published protocol [11].

### Testicular cell isolation

Testicular tissues were obtained by necropsy from the humanely euthanized fertile adult boars retired from the IVF semen collection program at the National Swine Research Resource Center (NSRRC, University of Missouri, Columbia, MO). Spermatocytes, spermatids and testicular spermatozoa were collected as described [12]. Briefly, tissues were dissected and processed to obtain a testicular cell suspension. Tissue fragments were transferred into TL-HEPES-PVA and gently dissociated to release the testicular cells. The resulting suspension was collected and subjected to centrifugation at 200 × g for 6 min, the supernatant was removed, and the step was repeated twice to remove debris. The final cell suspension containing male germ cells at different developmental stages was fixed in 2% formaldehyde in PBS for immunofluorescence as will be describe under Immunocytochemistry.

### Collection and in vitro maturation (IVM) of porcine oocytes

Porcine ovaries were obtained weekly from a local slaughterhouse. Cumulus-oocyte complexes (COCs) were aspirated from antral follicles of 2–6 mm size and collected into 50 mL conical tubes. The collected porcine follicular fluid was then diluted in HEPES buffered Tyrode Lactate medium containing 0.01% (*w/v*) polyvinyl alcohol (TL-HEPES-PVA) and searched for COCs. These COCs were then washed three times in TL-HEPES-PVA and transferred into 500 µL wells of oocyte maturation medium (OMM) (TCM 199; Mediatech, Inc., Manassas, VA) supplemented with 0.1% PVA, 3.05 mM D-glucose, 0.91 mM sodium pyruvate, 20 µg/mL gentamicin, 0.57 mM cysteine, 0.5 µg/mL LH (L5269, Sigma), 0.5 µg/mL FSH (F2293, Sigma), 10 ng/mL epidermal growth factor (E4127, Sigma), and 10% (*v/v*) porcine follicular fluid. The media was then covered with mineral oil in four-well dishes (Nunc, Roskilde, Denmark) and the COCs were incubated at 38.5 °C, 5% (v/v) CO_2_ in the air, for 43 h.

### In vitro fertilization (IVF) of porcine oocytes and in vitro culture (IVC) of zygotes

Cumulus cells of matured COCs were removed with 0.1% (*w/v*) hyaluronidase in TL-HEPES-PVA medium. These denuded oocytes were placed into oocyte maturation medium (OMM) and searched for MII oocytes, identified by the presence of a polar body. Quality parameters such as the presence of a dark solid metaphase chromosomes and correct morphology were also searched for. Mature oocytes were then washed three times with TL-HEPES-PVA medium and once with Tris-buffered medium (mTBM) containing 0.3% (*w/v*) BSA (A7888, Sigma). Between 20 and 40 oocytes were placed into 100 µL drops of the mTBM covered with mineral oil in a 35 mm polystyrene culture dish, then incubated until spermatozoa were prepared for fertilization. Liquid semen spermatozoa preserved in *Preserv® Xtreme* (GenePro) extender solution were washed with PBS containing 0.1% (w/v) PVA (PBS-PVA) two times by centrifugation at 450 × g for 5 min. To stain mitochondria in the sperm tail, the boar spermatozoa were incubated with vital, fixable, mitochondrion-specific probe MitoTracker® Red CMXRos for 10 min at 38.5 °C. The spermatozoa pre-labeled with MitoTracker were resuspended in mTBM medium and left to sit at room temperature for 5 min in the dark. The sperm suspension in mTBM medium was then added to the 100 µL drops of mTBM medium for a final concentration of 2.5 to 5 × 10^5^ spermatozoa/mL. Mature oocytes were incubated with spermatozoa for 5 to 6 h at 38.5 °C, 5% (v/v) CO_2_ in the air, then transferred to 500 µL drops of MU3 medium [13] containing 0.4% (w/v) BSA (A6003; Sigma) for additional culture.

### Sperm priming for cell-free system

Boar spermatozoa were centrifuged at 450 × g for 5 min and washed twice with phosphate-buffered saline (PBS, 137 mM NaCl, 2.7 mM KCl, 10 mM, 11 Na_2_HPO_4_, 1.8 mM KH_2_HPO_4_, pH = 7.2) containing 0.1% (w/v) PVA (PBS-PVA) To stain sperm mitochondria, the spermatozoa were pre-labeled with MitoTracker® Red CMXRos for 10 min at 38.5 °C. At 400 nM, the probe stains boar sperm mitochondria, but also may be taken up weakly by the sperm head structures [5]. In order to mimic natural fertilization, sperm mitochondria were demembranated and S–S were reduced [14]. Demembranation/permeabilization was performed by using 0.05% (*w/v*) lysolecithin (L-α-lysophosphatidylcholine, Sigma, St. Louis, MO, USA) in KMT (20 mM KCl, 5 mM MgCl_2_, 50 mM TRIS∙HCl, pH = 7.0) for 10 min at 37 °C. These primed spermatozoa were then washed twice with KMT for 5 min by centrifugation, to terminate the reaction. The spermatozoa were then incubated with 2.0 mM dithiothreitol (DTT; disulfide bonds/S–S reduction agent; Sigma, St. Louis, MO, USA) diluted in KMT, pH = 8.2 for 20 min at 37 °C and washed twice with KMT Ph = 8.2 for 5 min, to terminate the priming reaction.

### Preparation of porcine oocyte extracts and co-incubation with primed boar spermatozoa

Cumulus cells of matured COCs were removed with 0.1% (w/v) hyaluronidase in TL-HEPES-PVA medium. The oocytes were then searched for mature MII oocytes as designated by the presence of a polar body. Mature oocytes were then washed three times with TL-HEPES-PVA medium. Zonae pellucidae (ZP) were removed by pronase (0.1%, *w/v*) in TL-HEPES-PVA. The ZP-free, mature MII oocytes were transferred into an extraction buffer (50 mM KCl, 5 mM MgCl_2_, 5 mM ethylene glycol-bis(β-aminoethyl ether)-N,N,N’,N’-tetraacetic acid (EGTA), 2 mM β-mercaptoethanol, 0.1 mM PMSF, protease inhibitor cocktail (cat# 78410, ThermoFisher Scientific, Houston, TX), 50 mM HEPES, pH = 7.6) containing an energy-regenerating system (2 mM ATP, 20 mM phosphocreatine, 20 U/mL creatine kinase, and 2 mM GTP), and submerged three times into liquid nitrogen for 5 min each. Next, the frozen-thawed oocytes were crushed by high-speed centrifugation at 16,500 × g for 20 min at 4 °C in a Sorvall Biofuge Fresco (Kendro Laboratory Products). Batches of oocyte extract were made from 1,000 oocytes in 100 µL of extract. The supernatants were harvested, transferred into a 1.5 mL microcentrifuge tube, and stored in a deep freezer (–80 °C). Porcine oocyte extract was then added to the permeabilized boar spermatozoa at a concentration of 1 × 10^4^ spermatozoa/10 μL of an extract. They were then co-incubated in an incubator at 38.5 °C, with 5% (v/v) CO_2_ in the air for 4 or 24 h. After the determined co-incubation period, spermatozoa were washed three times with KMT. At this point, the spermatozoa were processed for either immunocytochemistry or electrophoresis.

### Immunocytochemistry

Oocytes, embryos, or spermatozoa were fixed in 2% (*v/v*) formaldehyde for 40 min at room temperature, washed in PBS, then processed. Oocytes could have also been stored in (0.1%, *w/v*) PBS-NaN_3_ at 4 °C until used for immunocytochemistry. If needed, some oocytes or embryos had their zona pellucida removed by using pronase (0.1%, *w/v*) in TL-HEPES-PVA, and the cells were then fixed in 2% (v/v) formaldehyde for 40 min at room temperature. To fix boar spermatozoa, whether ejaculated, primed, or CFS treated, microscopy coverslips were overlaid with 300 µL of 1% (*w/v*) poly-L-lysine in ultrapure water, incubated for 5 min, then shaken off and allowed to dry. The poly-L-lysine coated coverslips were overlaid with 400 µL of warm KMT medium (38.5 °C; pH 7.3) and 2 µL of sperm pellet were added onto coverslips and allowed to settle on the lysine-coated surface for 10 min on a 38.5 °C plate. The coverslips with spermatozoa were shaken off and transferred to 2% (v/v) formaldehyde for 40 min fixation at room temperature, then used for immunocytochemistry immediately or stored at 4 °C.

Both spermatozoa and oocytes were permeabilized in PBS with 0.1% (*v/v*) Triton-X-100 (PBS-TX) at room temperature for 40 min, then blocked in PBS-TX containing 5% (*v/v*) normal goat serum (NGS) for 30 min. The treated oocytes and spermatozoa were incubated overnight at 4 °C with primary antibodies diluted in PBS containing 1% (v/v) NGS and 0.1% (v/v) Triton-X-100. The samples were incubated with a secondary antibody solution including 2.5 µg/mL DNA stain DAPI (1:80 dilution), and goat anti-rabbit (GAR)-IgG-FITC (1:100 dilution), GAR-IgG-TRITC (1:100), goat anti-mouse (GAM)-IgG-FITC (1:100), or GAM-IgG-TRITC (1:100) for 40 min at room temperature (all fluorescently conjugated antibodies were purchased from Zymed/Thermo Fisher Scientific). Microscopy slides were labeled according to treatment, and the samples were then mounted on microscopy slides in VectaShield mounting medium (Vector Laboratories, Burlingame, CA). These slides were then photographed and analyzed with a Retiga QI-R6 camera (Teledyne QImaging, Surrey, BC, Canada) operated by MetaMorph 7.10.2.240. software (Molecular Devices, San Jose, CA). For zygote imaging, 15–30 IVF zygotes were used per replicate per time point (5, 15 and 25 h after IVF) in 3–4 replicates for each target protein, including an antibody labeled group and a negative control group. In addition, 8–10 eggs per treatment were used for antibody titration and validation trials in which 8–10 eggs were used per antibody.

### SDS-PAGE and Western blotting

Sperm proteins were extracted as described in [10] and mixed with 4 × LDS loading buffer (106 mM Tris∙HCl, 141 mM Tris base, 2% (*w/v*) lithium dodecyl sulfate, 10% (*v/v*) glycerol, 0.75% (*w/v*) Coomassie Brilliant Blue (CBB) G-250, 0.025% (*w/v*) Phenol Red, pH = 8.5) in 3:1 ratio, supplemented with 2.5% β-mercaptoethanol and incubated at 70 °C for 10 min. Spermatozoa were suspended in 1 × LDS loading buffer supplemented with 2.5% β-mercaptoethanol and incubated for an hour on a rocking platform at room temperature, then spun. Equal numbers of spermatozoa (5 to 10 million per lane), and protein extract (the equivalent of 20 µg per lane) were loaded in each lane on a NuPAGE 4–12% Bis–Tris gel (Invitrogen, Waltham MA). Electrophoresis was carried out in Bis–Tris system using MOPS-SDS running buffer (50 mM MOPS, 50 mM Tris base, 0.1% (*w/v*) SDS, 1 mM EDTA, pH = 7.7) with the cathode buffer supplemented with 5 mM sodium bisulfite. PAGE was carried out for 5 min at 90 Volts to let the samples delve into the gel, then the gel was allowed to run for another 60–70 min at 200 Volts. The power limit was set to 20 Watts. After PAGE, proteins were electro-transferred to polyvinylidene fluoride (PVDF) membranes (Millipore) by using Owl wet transfer system (Fischer Scientific) at 65 Volts for 90 min for immunodetection, using Bis–Tris-Bicine transfer buffer (25 mM Bis–Tris base, 25 mM Bicine, 1 mM EDTA, pH = 7.2) supplemented with 10% (*v/v*) methanol, and 2.5 mM sodium bisulfite. The molecular masses of separated proteins were estimated using Novex® Sharp Pre-stained Protein Standard (cat # LC5800, Invitrogen, Carlsbad, CA) run in parallel. The membranes with the transferred proteins were blocked with 10% (*w/v*) non-fat milk in TBS with 0.1% (*v/v*) Tween 20 (TBST; Sigma-Aldrich) and left to rock sedately for 40 min. The membranes were then left on a rocker and incubated at 4 °C, overnight, with the primary antibodies diluted 1:1,000. The next day, the membranes were incubated with the HRP-conjugated goat anti-mouse IgG (GAM-IgG-HRP diluted 1:10,000), or goat anti-rabbit (GAR-IgG-HRP diluted 1:10,000) as secondary antibodies (Thermo Fisher Scientific) for 40 min on a rocker, at room temperature. The membranes reacted with chemiluminescent substrate (Millipore Corporation, Billerica, MA), detected using ChemiDoc Touch Imaging System (Bio-Rad, Hercules, CA, USA) to record the protein bands, and analyzed by Image Lab Software (ver. 5.2.1, Bio-Rad, Hercules, CA, USA). The membranes were stained with CBB R-250 after chemiluminescence detection for protein load control.

#### STRING interactome analysis

Protein interactome analysis was conducted at string-db.org. Inputs included “multiple proteins by names/identifiers”, and separately analyzed species/organisms (*Sus scrofa, Homo sapiens, Mus musculus).* Outputs were saved as vector graphics files (.svg), and rasterized and saved as portable network graphic files (.png) by Adobe Photoshop 2025.

## Results

### Phenotyping of candidate proteins in the porcine cell-free system and zygotes

#### Phenotyping strategy

The candidate pro-autophagic proteins from the mass spectrometry results selected for further investigation include LACTB that functions by forming stable filaments to create intramitochondrial membrane organization within the mitochondria; peroxiredoxin 3 (PRDX3) which acts as a peroxide reducing agent and is necessary for normal mitochondrial function; proteasomal subunit alpha 8 (PSMA8) which is crucial for proper meiotic exit by the forming spermatids and is carried over into fully differentiated spermatozoa; translocase of outer mitochondrial membrane 34 (TOMM34) which imports pre-proteins into the mitochondria, as well as FUN14 domain containing 1 (FUNDC1) which functions as a mitophagy receptor that contributes to mitochondrial quality control following stress. These proteins of interest were further studied and validated by using IVF protocols, cell imaging, proteomics, and the aforementioned porcine CFS. Immunocytochemistry was used to describe the localization patterns of these candidate proteins in ejaculated (non-capacitated), primed, and cell-free-treated spermatozoa. All antibodies were validated by Western blotting of sperm extracts. Immunocytochemistry was also used to observe the localization patterns of these proteins in the in vitro derived porcine zygotes. Mature MII oocytes were fertilized with spermatozoa which were pre-labeled with MitoTracker so that the sperm mitochondrial sheaths could be detected after fertilization. The zygotes were then collected at 5-, 15-, and 25-h post-insemination (sperm and oocyte co-incubation). The early time points were selected to ensure that post-fertilization sperm mitophagy was proceeding as expected in the zygotes at both timepoints. An extra hour was added to 4-h and 24-h intervals applied to CFS studies, to account for the time required for sperm-zona pellucida adhesion and penetration during IVF. The 15-h time point was added because at this point, advancing but not yet completed sperm mitophagy is observed. Likewise, the 25-h time point was selected to observe the advanced and terminal stages of mitophagy post-fertilization. These presumed zygotes were then fixed and stained for immunocytochemistry.

#### Lactamase beta (LACTB)

**Fig. 1:**
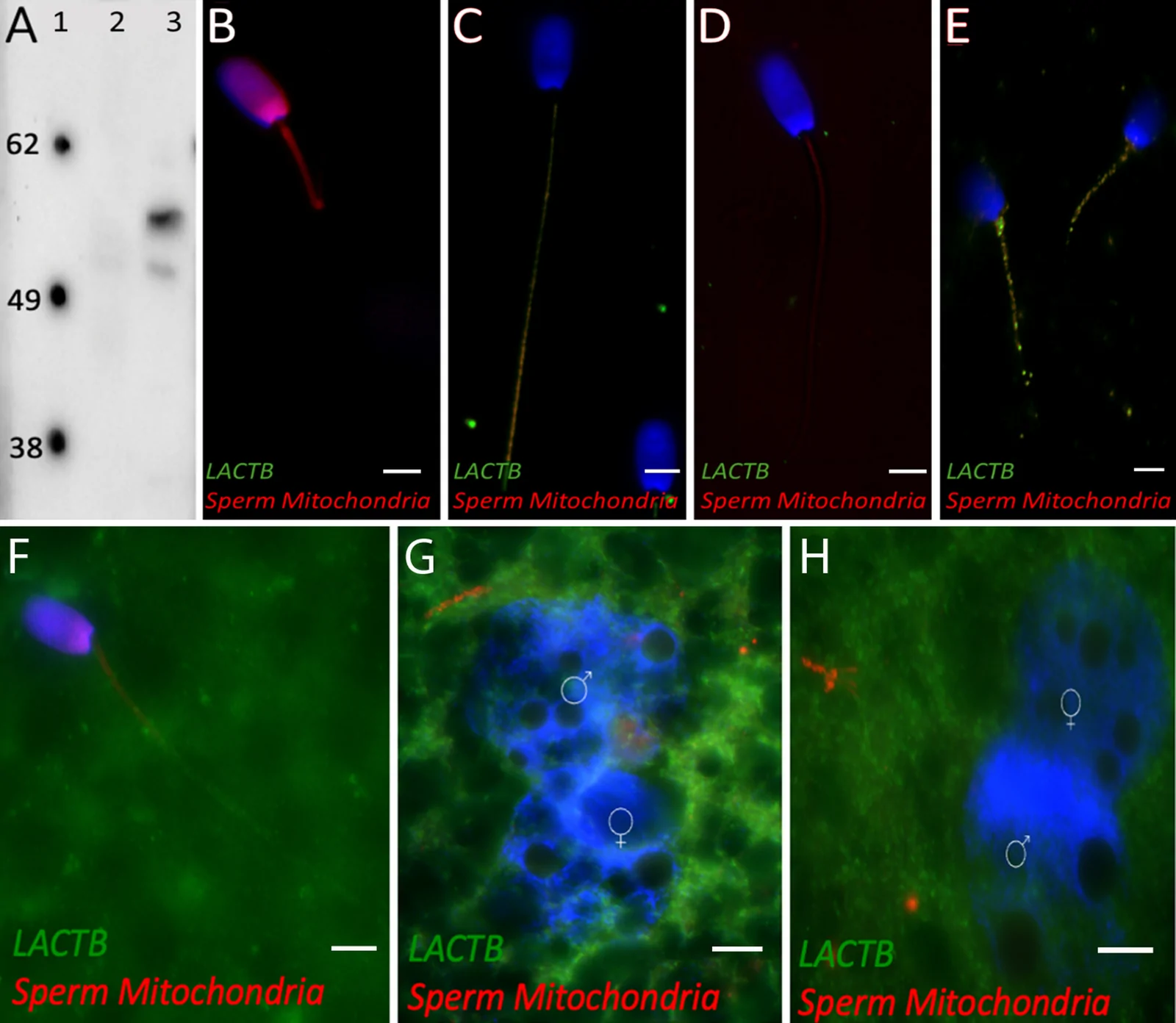
Lactamase beta in the porcine spermatozoa before and after capacitation and exposure to cell-free system. **A** By Western blotting, LACTB was below the limit of detection in ejaculated, non-capacitated spermatozoa (Lane 2), but it was detected at low level in capacitated spermatozoa (Lane 3) (molecular weight markers are shown in Lane 1; predicted mass ~ 54 kDa). LACTB was not detected in ejaculated, non-capacitated spermatozoa (**B**) or 4-h cell-free system exposed spermatozoa (**D**) via ICC using specific primary antibody anti-LACTB (PA5-57,627) and GAR-IgG-FITC (green). After priming (**C**) and 24-h exposure to the cell-free system (**E**), LACTB (green) was detected throughout the tail of the treated spermatozoa. The mitochondrial sheath (MS-red) was labeled by MitoTracker. DNA was counterstained with DAPI (blue). (**F**) In the 5-h post-insemination oocytes, LACTB (green) was detected abundantly by using specific primary anti-LACTB antibody (PA5-57,627) and GAR-IgG-FITC in the cytoplasm as well as along the entirety of the spermatozoa tail. **G, H** In zygotes 15 (**G**) and 25 h post-insemination (**H**), LACTB was still detected in the cytoplasm, although it did not appear to be associated with the paternal pronuclei or with the mitochondrial sheaths of the fertilizing spermatozoa. Sperm mitochondria (red) were labeled with MitoTracker, DNA was counterstained with DAPI (blue). Scale bars = 5 μm

Lactamase beta was identified as a Class 1 protein of interest in our mass spectrometry trials. It was not detected in vehicle control spermatozoa or primed control sperm samples. However, it was identified in the oocyte extracts and in spermatozoa exposed to the CFS at the 24-h time point, but not at the 4-h time point (see proteomic data in [10]). Upon further investigation, we confirmed that LACTB was not detectable in ejaculated, non-capacitated spermatozoa by using both Western blotting and immunocytochemistry detection methods (Fig. 1A, B). Inconsistent with the mass spectrometry results, the LACTB protein was detectable by Western blotting in the capacitated spermatozoa (Fig. 1** A**) and the immunofluorescence labeling of LACTB labeling was found along the tail of the spermatozoa after priming (Fig. 1C). However, consistent with the mass spectrometry results, LACTB was not detected after the 4-h CFS exposure (Fig. 1D). The labeling persisted in the tails of treated spermatozoa after 24 h of CFS exposure (Fig. 1E**)**. In the 5-h post-insemination oocytes, LACTB was detected abundantly in the cytoplasm as well as along the entirety of the sperm tail (Fig. 1F). This labeling pattern is consistent with the LACTB labeling found in primed spermatozoa as well as 24-h CFS treated spermatozoa. In zygotes at 15- and 25-h post-insemination, LACTB was still detectable in the cytoplasm, although it did not appear to be associated with forming paternal pronuclei (PPN) or with the mitochondrial sheaths of the fertilizing spermatozoa (Fig. 1G, H).

#### Peroxiredoxin 3 (PRDX3)

**Fig. 2:**
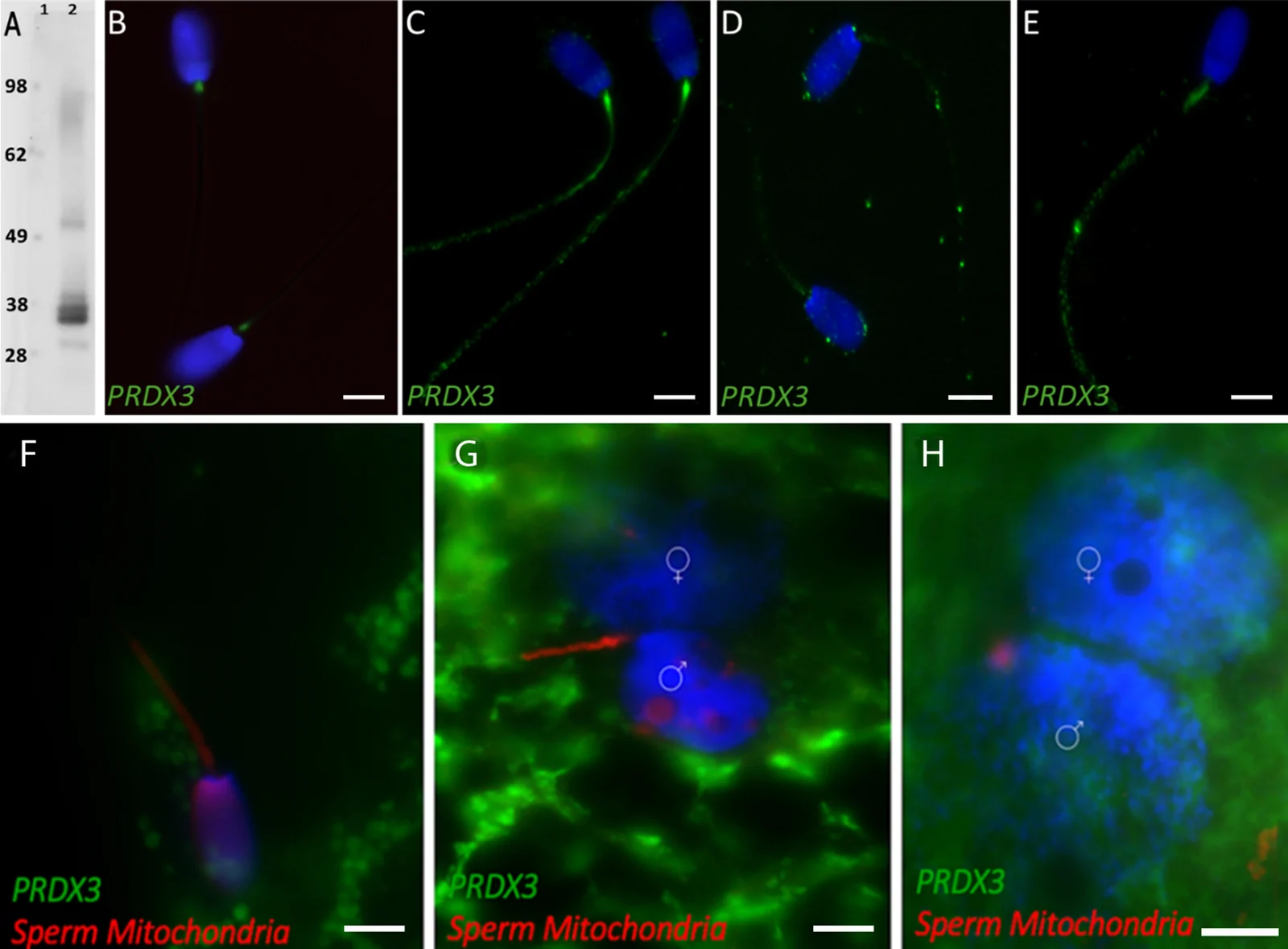
Peroxiredoxin 3 in the porcine spermatozoa, cell-free system and zygotes. (**A**) PRDX3 was detected in ejaculated, non-capacitated spermatozoa by using Western blotting (Lane 2) (molecular weight markers in Lane 1; predicted mass ~ 28 kDa), and immunocytochemistry (green) using specific primary antibody anti-PRDX3 (PIPA591918) and GAR-IgG-FITC (**B**), where it was found to localize to the connecting piece. (**C**) This connecting piece labeling persisted in primed spermatozoa with the addition of localization throughout the tail. (**D**) After 4 h of cell-free system exposure, PRDX3 was still located in the connecting piece as well as along the tail, but it was not as abundant. (**E**) After 24 h of cell-free system exposure, this PRDX3 labeling persisted in the connecting piece as well as along the tail, but it was more distinct. DNA was counterstained with DAPI (blue). (**F, G**) Labeling of PRDX3 (green) with a specific primary anti-PRDX3 antibody (PIPA591918) and GAR-IgG-FITC were not observed localizing on the fertilizing spermatozoa at 5 h (**F**) or 15 h post-insemination (**G**), though PRDX3 did appear to be present in the cytoplasm of the zygote. (**H**) In zygotes at 25 h post-insemination, PRDX3 was still not observed on the fertilizing spermatozoa, but remained present within the cytoplasm of the zygote. Sperm mitochondria (red) were labeled with MitoTracker, DNA was counterstained with DAPI (blue). Scale bars = 5 μm

Peroxiredoxin 3 was also identified as a Class 1 protein of interest in our mass spectrometry trials [10] wherein an insignificant amount of PRDX3 was detected in vehicle control spermatozoa or primed samples. However, it was identified by proteomics in the oocyte extracts and in spermatozoa exposed to the CFS at both the 4- and 24-h times points [10]. Upon further investigation, we found that PRDX3 protein was detectable in spermatozoa by Western blotting (Fig. 2A) in a multi-band pattern agreeing with published work (e.g., [15, 16]) and localized to the connecting piece of ejaculated, non-capacitated boar spermatozoa by using immunocytochemistry (Fig. 2B). Additionally, after priming the spermatozoa, PRDX3 was detected on the connecting piece as well as a faint signal along the tail (Fig. 2C). Consistent with MS observations, PRDX3 was detected in the connecting piece as well as the tails of treated spermatozoa after both 4 and 24 h of CFS exposure (Fig. 2D, E). The PRDX3 protein was not observed on the fertilizing spermatozoa at 5- or 15-h post-insemination, though PRDX3 was detected in the cytoplasm of the zygotes (Fig. 2F, G). In zygotes at 25 h post-insemination, PRDX3 was still not detectable on the fertilizing spermatozoa, but remained present within the cytoplasm of the zygote (Fig. 2H).

#### Proteasomal subunit alpha 8 (PSMA8)

**Fig. 3:**
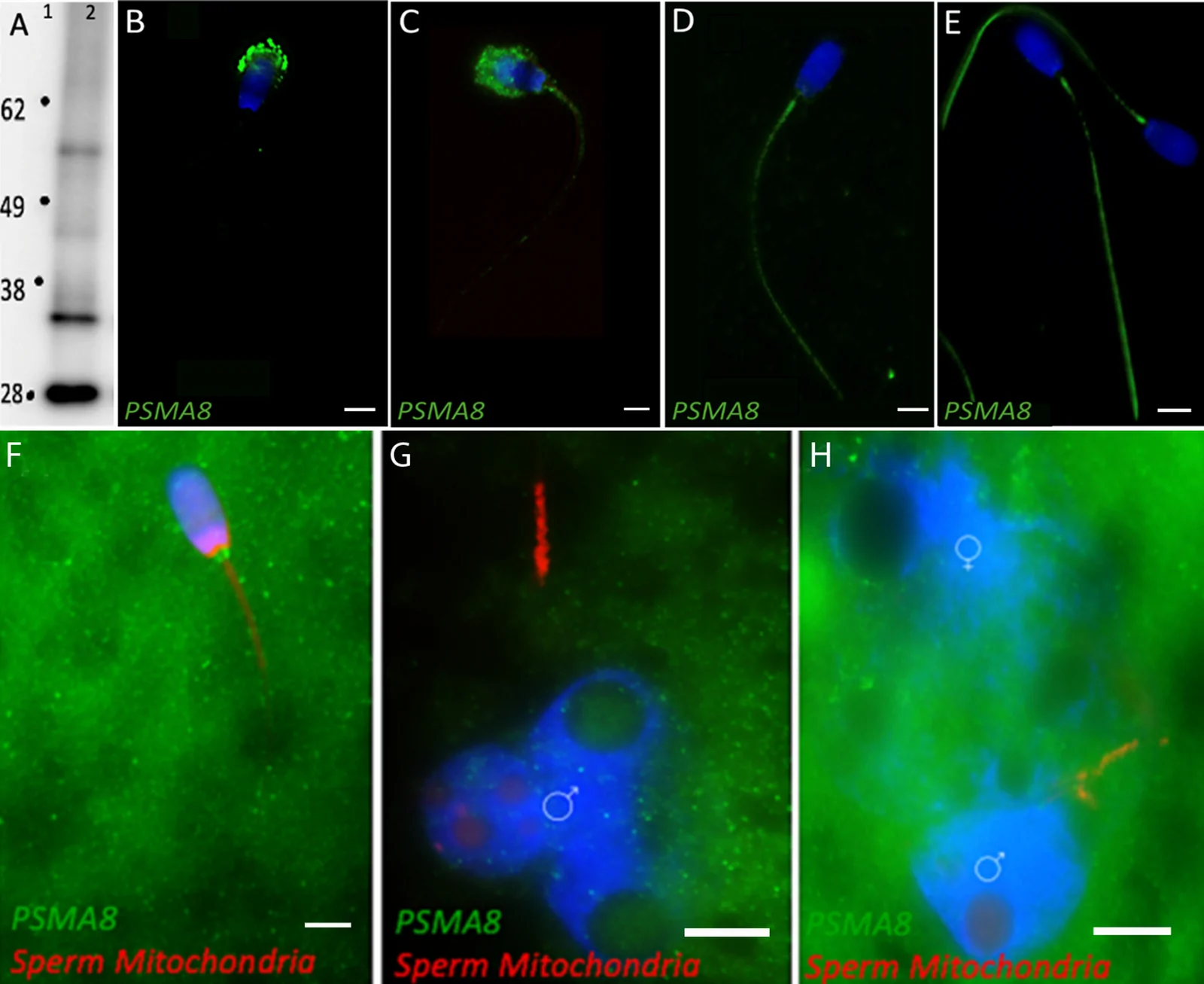
Proteasomal subunit alpha 8 in the porcine spermatozoa, cell-free system and zygotes. **A** PSMA8 was detected in ejaculated, non-capacitated spermatozoa via Western blotting (markers in Lane 1; predicted mass ~ 28 kDa), and immunocytochemistry (green) using specific primary antibody anti-PSMA8 (50–172-9159) and GAR-IgG-FITC (**B**), where it was found to localize to the acrosome. (**C**) In primed spermatozoa, PSMA8 was not only detected in the acrosome of the spermatozoa but also in the mitochondrial sheath. (**D**) After 4 h of cell-free system co-incubation, PSMA8 was no longer detected in the head of the spermatozoa, but it was detected along the full length of the tail. (**E**) After 24 h of CFS exposure, this labeling persisted but with a more distinct labeling in the connecting piece as well as the principal piece of the sperm tail. DNA was counterstained with DAPI (blue). (**F**) In the 5-h post-insemination oocytes, PSMA8 labeling (green) with specific primary anti-PSMA8 antibody (50–172-9159) and GAR-IgG-FITC were detected in the cytoplasm as well as on the head, connecting piece and along the tail of the fertilizing spermatozoa. (**G**) In zygotes 15 h post-insemination, PSMA8 was detected abundantly in the cytoplasm and appeared to be associating with forming paternal pronuclei, but not the mitochondrial sheath. (**H**) In zygotes 25 h post-insemination, PSMA8 was still detected in the cytoplasm, as well as associating with forming pronuclei, but no association was observed in the mitochondrial sheaths of the fertilizing spermatozoa. Sperm mitochondria (red) were labeled with MitoTracker, DNA was counterstained with DAPI (blue). Scale bars = 5 μm

During the quantitative mass spectrometry trials, proteasomal subunit alpha 8 was identified as a Class 2 protein. The relative abundance of PSMA8 increased significantly in the 4-h CFS trial (*p* = 0.09) as well as 24-h trial (*p* = 0.05) when compared to the primed control spermatozoa [10]. The PSMA8 protein was detected in the ejaculated, non-capacitated spermatozoa at its expected weight of 28 kDa, with additional minor bands [17], via Western blotting (Fig. 3A). It was also found to localize to the acrosome of ejaculated, non-capacitated spermatozoa via immunocytochemistry (Fig. 3B). After priming, PSMA8 was still detectable in the head of the spermatozoa, in addition to the mitochondrial sheath localization (Fig. 3C). After 4 h of CFS co-incubation, PSMA8 was no longer detected in the head of the spermatozoa, but it was detected along the full length of the tail (Fig. 3D). After 24 h of CFS exposure, this labeling persisted but with a brighter signal in the connecting piece as well as the principal piece of the sperm tail (Fig. 3E). Some but likely not all PSMA8 signal loss from the sperm head could be attributed to sperm acrosome damage during the priming procedure prior to CFS. In the 5-h post-insemination oocytes, PSMA8 was detected in the cytoplasm as well as on the head, connecting piece, and along the tail of the fertilizing spermatozoa (Fig. 3F). This labeling was like the PSMA8 labeling observed in the primed, 4-h and 24-h CFS treated spermatozoa. In zygotes 15 h post-insemination, PSMA8 was detected abundantly in the cytoplasm and appeared to be associated with forming paternal pronuclei, but not the mitochondrial sheath (Fig. 3G). In zygotes 25 h post-insemination, PSMA8 was still detectable in the cytoplasm, as well as associating with forming pronuclei, but no association was observed with the mitochondrial sheaths of the fertilizing spermatozoa (Fig. 3H).

#### Translocase of outer mitochondrial membrane 34 (TOMM34)

**Fig. 4:**
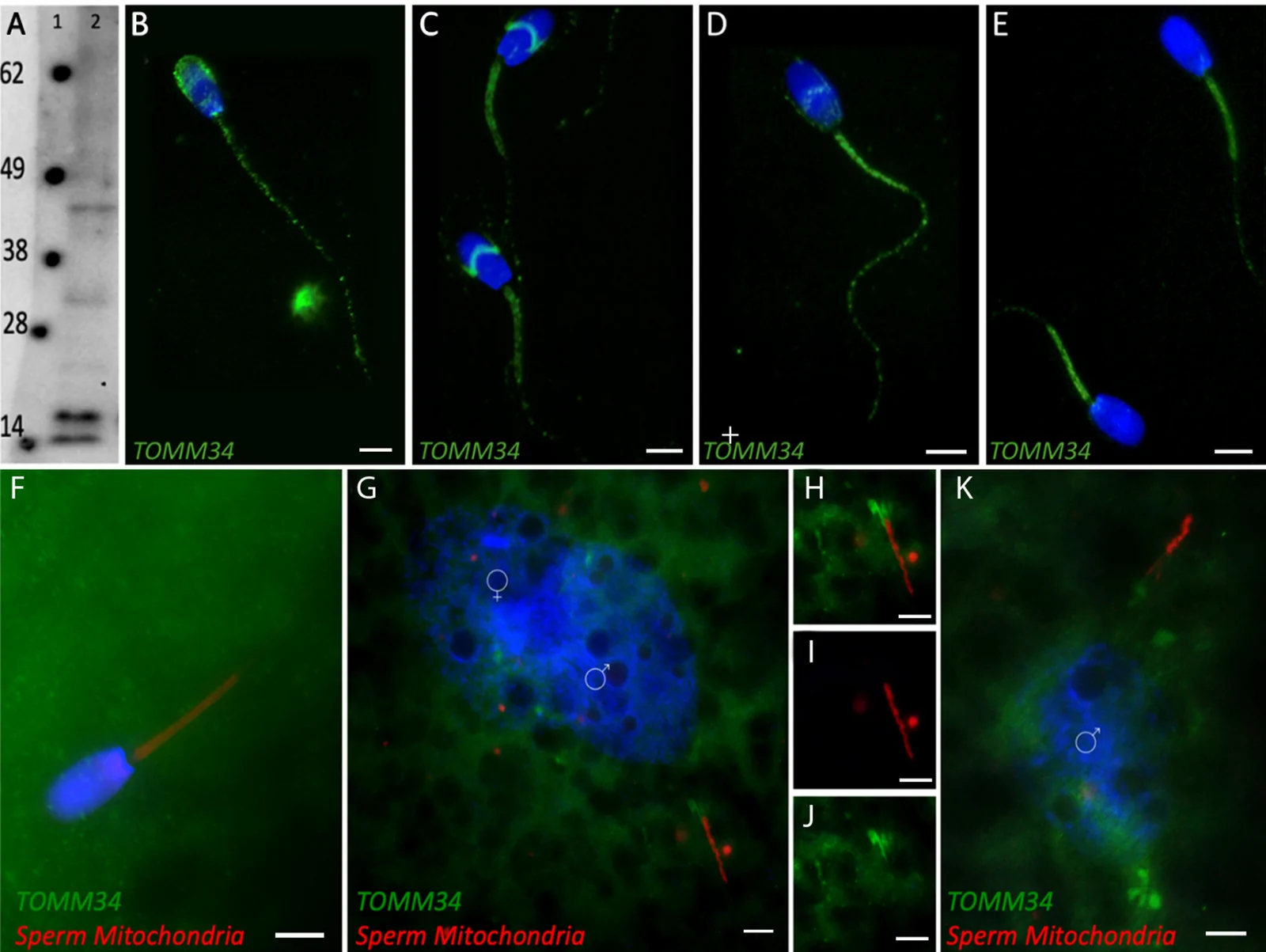
Translocase of outer mitochondrial membrane 34 in the porcine spermatozoa, cell-free system and zygotes. **A** TOMM34 was detected in ejaculated, non-capacitated spermatozoa by using Western blotting (markers in Lane 1; predicted mass =  ~ 34 kDa), and immunocytochemistry (green) using specific primary antibody anti-TOMM34 (NBP220682) and GAR-IgG-FITC (**B**), where it was found to be localized to the acrosome as well as along the length of the tail. (**C**) After priming, TOMM34 was detected in the equatorial segment as well as the mitochondrial sheath. **D** At 4 h after exposure to the cell free system, TOMM34 was detected in the equatorial segment, the principal piece, and in the mitochondrial sheath. **E** After 24 h of cell free system exposure, TOMM34 localization was confined to the mitochondrial sheath. DNA was counterstained with DAPI (blue). **F** Labeling of TOMM34 (green) with a specific primary anti-TOMM34 antibody (NBP220682) and GAR-IgG-FITC were observed in the 5-h post-insemination oocytes in the cytoplasm but were not detected on the fertilizing spermatozoa. When observing the zygotes 15 (**G–J**) and 25 h post-insemination (**K**), TOMM34 was still detected in the cytoplasm, but there also appeared to be a clustering of TOMM34 on the proximal end of the fertilizing spermatozoa’s mitochondrial sheath, though it did not appear to be associated with pronuclei. (**H**) A zoomed in cutout of the MS.The green/protein labeling channel separation is shown in (**J**). The MS is shown by MitoTracker labeling in red channel separation panel (I). **K** The 25-h post-insemination zygotes still had the FUNDC1 labeling in the cytoplasm, but there was no association of FUNDC1 with the pronuclei or the mitochondrial sheath of the fertilizing spermatozoa. Sperm mitochondria (red) were labeled with MitoTracker; DNA was counterstained with DAPI (blue). Scale bars = 5 μm

Translocase of outer mitochondrial membrane 34 was classified as a Class 3 protein in our mass spectrometry trials. In agreement with the mass spectrometry classification, TOMM34 was observed to undergo a significant decrease in abundance after 4 h of CFS exposure (*p* = 0.031). No significant change was observed in the abundance of TOMM34 during the 24-h trial when compared to the primed control spermatozoa. TOMM34 underwent a reduction in all three of the mass spectrometry trial replicates [10]. In Western blotting, TOMM34 was detected at its expected weight of 34 kDa as well as at 45 kDa in ejaculated, non-capacitated boar spermatozoa (Fig. 4A). By using immunocytochemistry, TOMM34 was detected in the acrosome as well as along the entire length of sperm tail (Fig. 4B). After priming, TOMM34 was no longer detected in the acrosome of the spermatozoa, possibly due to acrosomal membrane damage caused by priming. Inversely, TOMM34 became detectable in the equatorial segment of the sperm head as well as the mitochondrial sheath after priming (Fig. 4C). Four hours after exposure to the CFS, TOMM34 was detected lightly in the equatorial segment as well as the principal piece and distinctively in the mitochondrial sheath (Fig. 4D**)**. After 24 h of CFS exposure, TOMM34 localization was confined to the mitochondrial sheath (Fig. 4E). The TOMM34 protein was observed in the 5-h post-insemination oocytes in the cytoplasm but was not detected on the fertilizing spermatozoa (Fig. 4F). When observing the zygotes 15- and 25-h post-insemination, TOMM34 was still detected in the cytoplasm. There also appeared to be a clustering of TOMM34 on the proximal end of the fertilizing spermatozoa’s mitochondrial sheath (Fig. 4G-K). However, it did not appear to be associated with forming pronuclei (Fig. 4G, K).

#### FUN14 domain containing 1 (FUNDC1)

**Fig. 5:**
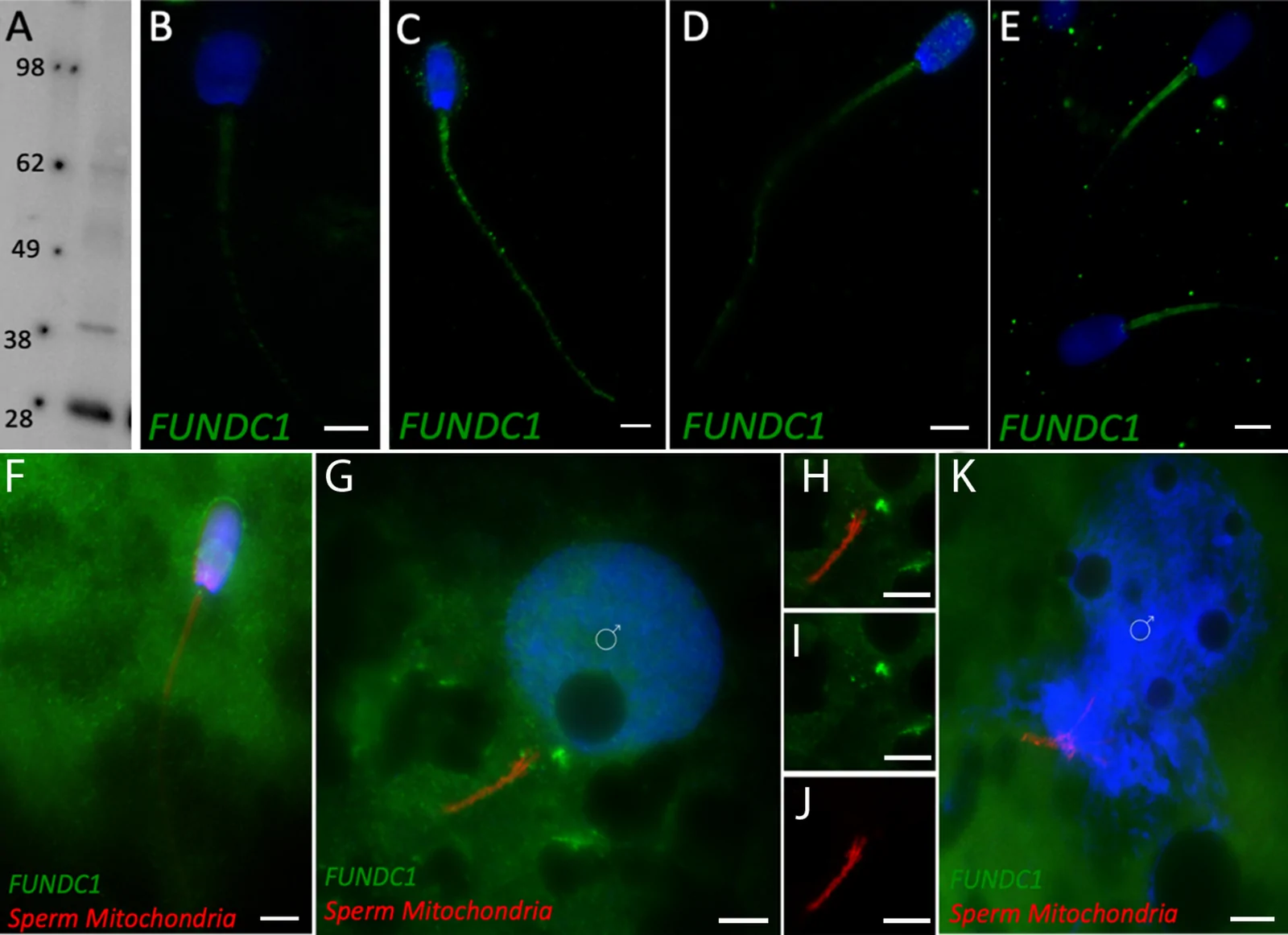
Patterns of FUN14 domain containing 1 in the porcine spermatozoa, cell-free system and zygotes. **A** A larger than expected FUNDC1 protein band was detected in the ejaculated, non-capacitated spermatozoa via Western blotting (markers in Lane 1; predicted mass ~ 17 kDa) as well as in the mitochondrial sheath of ejaculated, non-capacitated spermatozoa by immunocytochemistry (green) using specific primary antibody against FUNDC1 (ab224722) and GAR-IgG-FITC (**B**). **C** After priming, FUNDC1 was detected on the head of the spermatozoa as well as throughout the tail, with a stronger signal in the mitochondrial sheath. **D** After 4 h of exposure, the signal persisted along the tail of the spermatozoa as well as in the mitochondrial sheath. **E** After 24 h of CFS exposure, FUNDC1 was confined to the mitochondrial sheath of the spermatozoa. DNA was counterstained with DAPI (blue). **F** In the 5-h post-insemination oocytes, FUNDC1 (green) was detected by using specific primary anti-FUNDC1 antibody (ab224722) and GAR-IgG-FITC in the cytoplasm as well as on the acrosome of the fertilizing spermatozoa. **G** After 15 h post-insemination, FUNDC1 was still found in the cytoplasm of the zygotes as well as associated with the paternal pronuclei. **H** A zoomed in cutout of the MS. **I** The green/protein labeling channel separation is shown. (**J**) The MS is shown by MitoTracker labeling in red channel separation panel. **K** The 25-h post-insemination zygotes still had the FUNDC1 labeling in the cytoplasm, but there was no association of FUNDC1 with the pronuclei or the mitochondrial sheath of the fertilizing spermatozoa. Sperm mitochondria (red) were labeled with MitoTracker, DNA was counterstained with DAPI (blue). Scale bars = 5 μm

The FUN14 domain containing 1 was not identified in our mass spectrometry data, but it is a known mitophagic protein and its homolog FUNDC2 was identified as a Class 3 protein during our mass spectrometry trials [10]. Therefore, we continued to study FUNDC1 in our porcine CFS. FUNDC1 was detected in ejaculated, non-capacitated spermatozoa via Western blotting (Fig. 5A) as well as in the mitochondrial sheath of ejaculated, non-capacitated spermatozoa by using immunocytochemistry (Fig. 5B). After priming, FUNDC1 was detected on the head of the spermatozoa as well as throughout the tail with a stronger signal in the mitochondrial sheath (Fig. 5C). After 4 h of exposure, the signal along the tail and mitochondrial sheath persisted, but FUNDC1 was no longer detected on the head of the spermatozoa (Fig. 5D). After 24 h of CFS exposure FUNDC1 was confined to the mitochondrial sheath of the spermatozoa (Fig. 5E). In the 5-h post-insemination oocytes, FUNDC1 was detected in the cytoplasm as well as on the acrosome of the fertilizing spermatozoa (Fig. 5F). This labeling is similar to the labeling observed on the head of the primed spermatozoa. After 15 h post-insemination, FUNDC1 was still found in the cytoplasm of the zygotes as well as associated with the forming paternal pronuclei, with some FUNDC1 positive particles located adjacent to the disintegrating mitochondrial sheaths (Fig. 5G-J). The 25-h post-insemination zygotes still had the FUNDC1 labeling in the cytoplasm, but there was no association of FUNDC1 with the pronuclei or the mitochondrial sheath of the fertilizing spermatozoa (Fig. 5K).

### Investigation of candidate proteins in the porcine round and elongating spermatids

#### Strategy

To examine their developmental origin, the 5 candidate sperm proteins were used for testicular cell immunocytochemistry (ICC). Previously fixed boar spermatogenic cells were adhered to coverslips and permeabilized, followed by a blocking solution, as described for sperm immunofluorescence. The appropriate primary and secondary antibody solutions were used to identify the individual target proteins of the various spermatogenic cell types including spermatocytes, round spermatids, early and late-step elongating spermatids, and the occasional immature spermatozoa. Fluorescent probes used included FITC-conjugated secondary antibodies (goat anti-rabbit-GAR/goat anti-mouse-GAM) for target protein labeling, lectin PNA-TRITC to identify the developing acrosome, and DAPI to counterstain the nucleus. Once ICC was complete, coverslips were mounted onto slides and viewed under a Nikon Eclipse 800 fluorescent microscope (Nikon Instruments Inc, Melville, NY).

#### Translocase of outer mitochondrial membrane 34 (TOMM34)

**Fig. 6:**
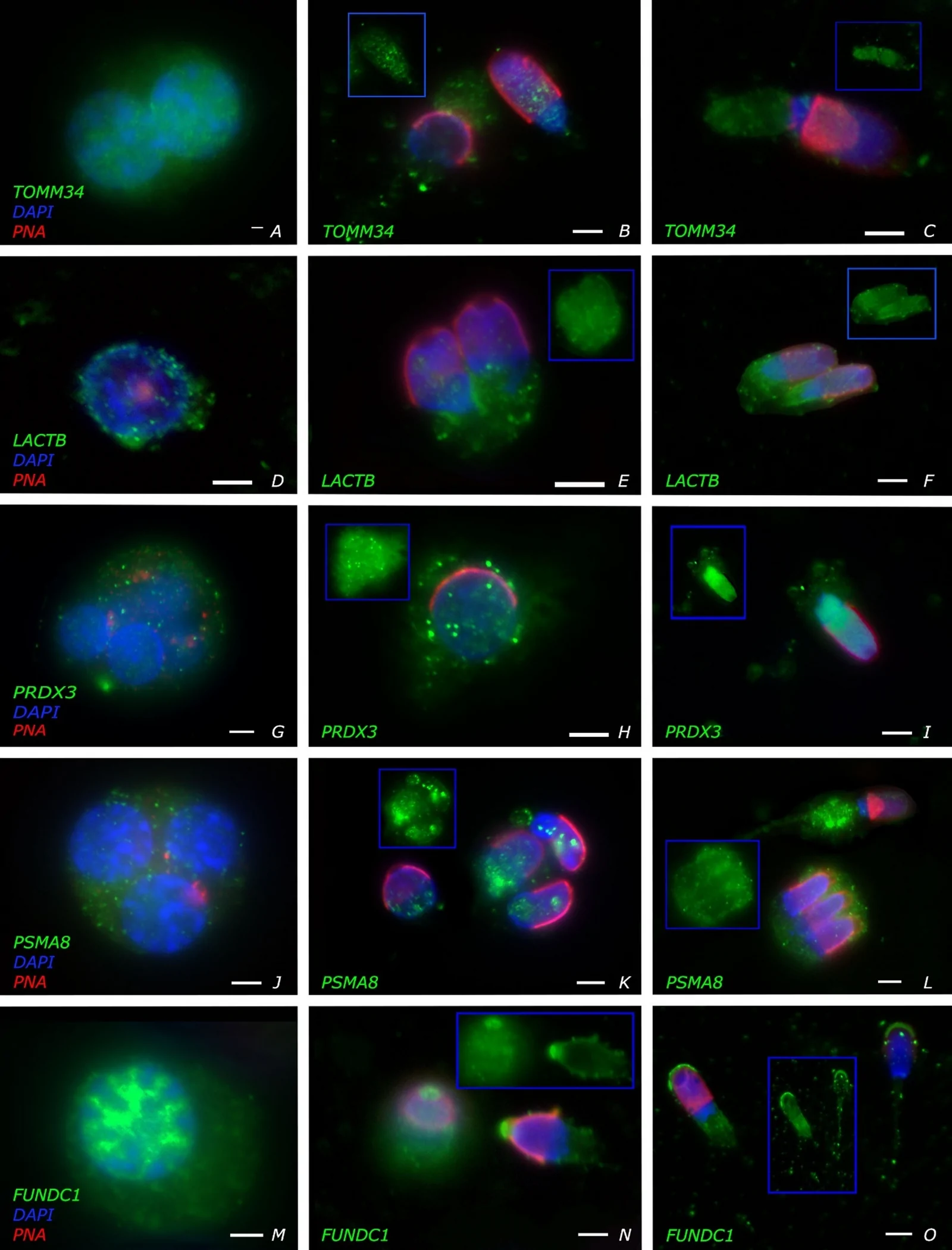
Immunocytochemistry of testicular germ cells provided visualization of the target proteins at various stages of spermatogenesis and steps of spermiogenesis. **A** The TOMM34 protein (green) was visualized within the cytoplasm of spermatocytes. **B** The signal persisted in the cytoplasm of round and early elongating spermatids, localizing faintly with the post-acrosomal region and developing connecting piece or centriole, as visible in the inset showing the TOMM34 signal alone. **C** As the spermatid elongation advanced, TOMM34 became weaker near the apical region, while stronger over the post-acrosomal region and cytoplasmic lobe/droplet and still present in the head. **D** The LACTB protein (green) was visualized within the cytoplasm of the spermatocytes. **E** The protein signal remained within the developing head and cytoplasm of the round spermatids. **F** In elongating spermatids, signals were localized to the outer acrosomal membrane, manchette, and the cytoplasmic lobe, as visible in the inset. **G** The PRDX3 protein (green) was visualized within the cytoplasm of the spermatocytes. **H** The signal intensified throughout the cytoplasm with the addition of granules/aggregates in round spermatids. **I** Through the elongation stages, PRDX3 can be seen in the head, with a stronger presentation in the post-acrosomal region. Protein signal decreased within the cytoplasmic lobes, as shown in the inset. **J** The PSMA8 protein (green) was visualized within the cytoplasm of the spermatocytes. The signal remained throughout the cytoplasm of round spermatids with the addition of granules. **K** There was a localization to the acrosomal membrane and post-acrosomal region. The PSMA8 can be visualized in the cytoplasm of elongating spermatids, with stronger signal on the outer edge of the acrosome (**K, L**). **L** In late-stage elongation and immature spermatozoa, there was a signal in the cytoplasm with an increase in signal intensity in the lobe/droplet and the developing mitochondrial sheath of the nascent tail. **M** The FUNDC1 protein (green) was visualized within the cytoplasm of the spermatocyte. The signal remained throughout the cytoplasm of round and early elongating spermatids. **N** The signal co-localized strongly with the acrosomal granule and outer acrosomal membrane. Through early and late elongation stages, FUNDC1 can be found within the acrosomal cap and cytoplasmic lobe. **O** The signal decreased overall as the spermatozoa completed elongation; FUNDC1 was visualized then over the mitochondrial sheath and tail and presented stronger to the acrosomal cap, as visible in the insets. Sperm acrosomes (red) were labeled with PNA-TRITC, DNA was counterstained with DAPI (blue). Scale bars = 5 μm

The TOMM34 protein was visualized within the cytoplasm of spermatocytes **(**Fig. 6A**)**. The signal remained throughout the cytoplasm of round and early elongating spermatids. The signal also localized faintly with the post-acrosomal region and developing connecting piece or centriole of early elongating spermatids **(**Fig. 6B**)**. In later elongation steps, TOMM34 faded within the apical region of the sperm head, while stronger signal was present over the post-acrosomal sheath and cytoplasmic lobe **(**Fig. 6C**)**.

#### Lactamase beta (LACTB)

The LACTB protein was visualized within the cytoplasm of the spermatocytes (Fig. 6D). The protein signal remained within the developing head of the round and elongating spermatids, with localization to the outer membrane, caudal manchette, and the cytoplasmic lobe (Fig. 6E, F).

#### Peroxiredoxin 3 (PRDX3)

The PRDX3 protein was visualized within the cytoplasm of the spermatocytes (Fig. 6G). The signal intensified and was seen throughout the cytoplasm and in granules of round spermatids (Fig. 6H). As the spermatids progressed through the elongation stages, PRDX3 could be seen in the head, with a stronger presentation in the post-acrosomal region; inversely, there was a fainter signal within the cytoplasmic lobes (Fig. 6I).

#### Proteasomal subunit alpha 8 (PSMA8)

The PSMA8 protein was visualized within the cytoplasm of the spermatocyte (Fig. 6J). The diffuse signal remained throughout the cytoplasm of round spermatids with the addition of dispersed PSMA8-contaning aggregates/granules. There was also a distinct localization to the acrosomal membranes and post-acrosomal region (Fig. 6K) as the spermatids began to elongate. Later during elongation, the PSMA8 remained present in the cytoplasm, with stronger signal on the outer edge of the developing acrosome and a dot-like pattern in the cytoplasmic lobe, reminiscent of mitochondrion distribution at the onset of mitochondrial sheath formation. In late stage elongated spermatids and immature spermatozoa, there was an even signal throughout the cytoplasm with an increase in the cytoplasmic droplet/lobe and tail midpiece of late step spermatid and spermatozoa (Fig. 6L).

#### FUN14 domain containing 1 (FUNDC1)

The FUNDC1 protein was visualized within the cytoplasm of the spermatocytes, with strong inter-chromosomal localization in pachytene secondary spermatocytes and retention thereof in very early post-meiotic spermatids with somewhat condensed chromosomes (Fig. 6M). The signal remained throughout the cytoplasm of round and early elongating spermatids. The signal co-localized strongly with the acrosomal granule and outer acrosomal membrane (Fig. 6N). Through the early and late elongation steps, FUNDC1 was found to localize to the acrosomal cap and the cytoplasmic lobe. The signal decreased overall as the spermatozoa completed elongation; however, FUNDC1 was then visualized over the mitochondrial sheath and tail principal piece, and more strongly present in the acrosomal cap (Fig. 6O).

### Interactome analysis

**Fig. 7:**
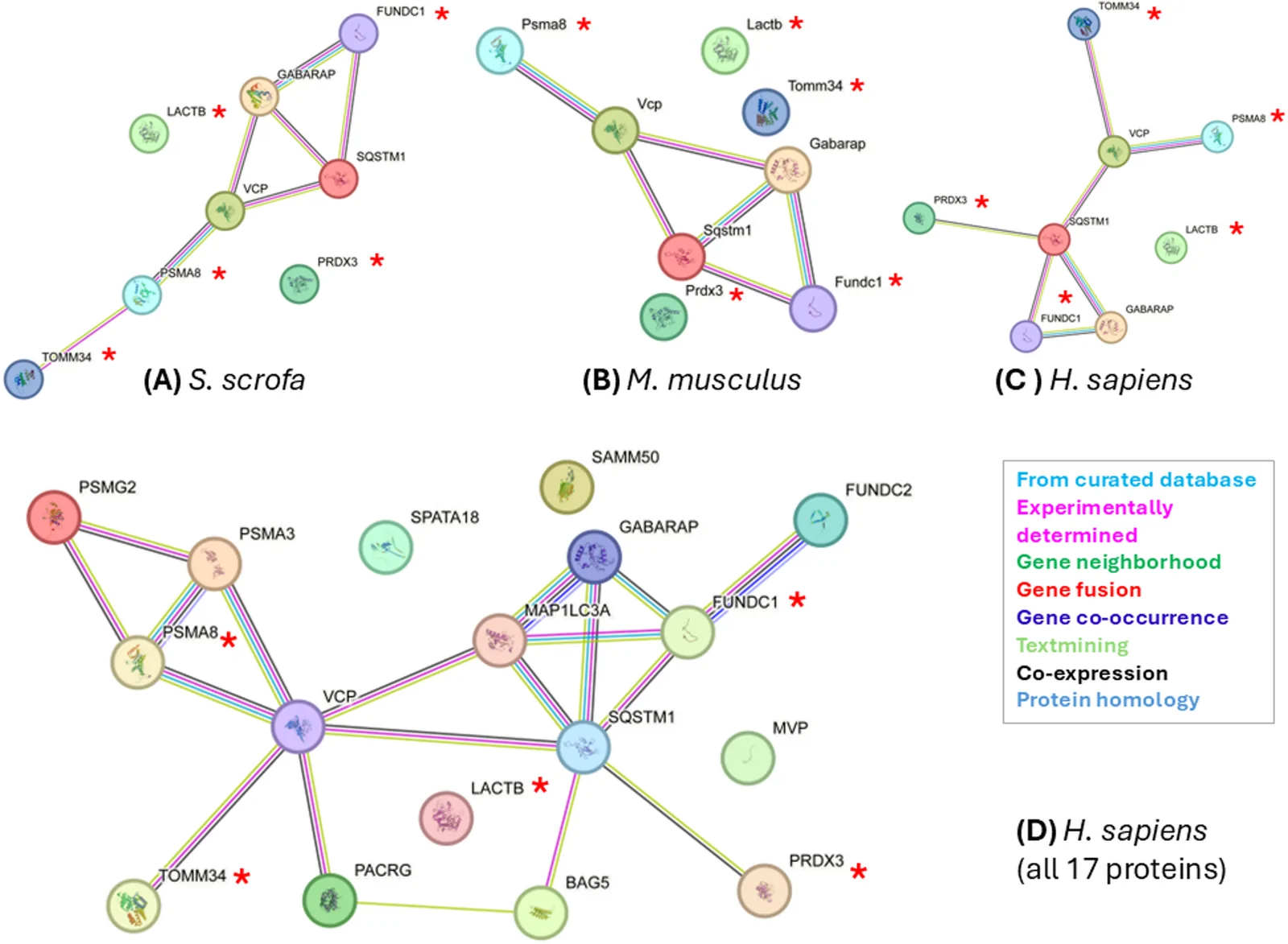
**A**–**C** The human, mouse and pig interactome networks of five proteins described in this study, centered around three key autophagic proteins, SQSTM1, VCP and GABARAP, experimentally tested as detailed in our published work. Three orphan (no connections) proteins are present in murine and porcine interactomes compared to only one in the human network, prompting the choice of human interactome as the primary resource for additional interactome analysis. **D** Summary interactions of 17 autophagy factor proteins phenotyped in our published and preliminary data (human interactome is shown). Red asterisks in all panels denote the positions of the five proteins phenotyped in this study

The STRING interactome analysis (string-db.org) revealed a striking clustering pattern of the five examined proteins (Fig. 7** A-C**), centered around three experimentally tested sperm mitophagy factors, VCP, GABARAP and SQSTM1 [5]. In addition to porcine interactome, the murine and human interactomes were examined because they are based on more comprehensive genomic and proteomic data sets. The only orphan protein in all three species’ interactomes was LACTB. The individual interactomes of five proteins in this study fit closely with the interactomes of 12 known sperm mitophagy autophagy factors phenotyped in our published studies (Fig. 7D), including SQSTM1, GABARAP, VCP and LC3/MAP1LC3A [5]; PACRG and SPATA18 [8]; and MVP, PSMG2, PSMA3, FUNDC2, SAMM50, and BAG5 [10]. In this protein set, FUNDC1 interacted closely with the major autophagy proteins (GABARAP, LC3A, and SQSTM1), and most strongly with its close relative, FUNDC2. Proteasomal core subunit PSMA8 clustered with two other proteasomal subunits (PSMA3 and PSMG2), as well as with the proteasome interacting valosine-containing protein (VCP), providing a link to LC3A and SQSTM1. Likewise, TOMM34 interacted with VCP while PRDX3 showed interaction with SQSTM1. Such interaction patterns indicate that at least four of the five examined proteins are involved in autophagy/mitophagy.

## Discussion

**Fig. 8:**
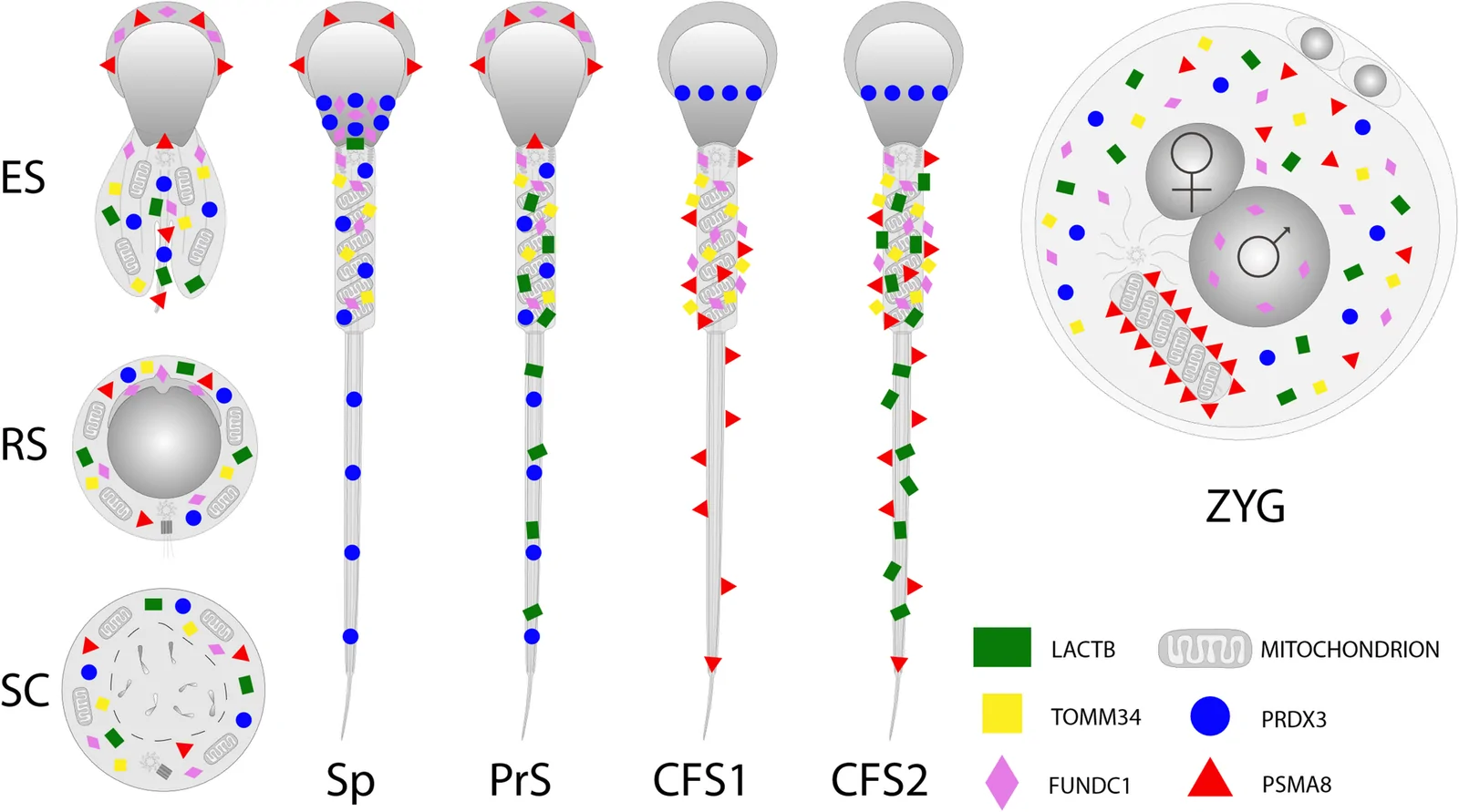
A stylized depiction of the candidate mitophagy proteins’ localization patterns in boar spermatids and spermatozoa during spermatogenesis, cell-free system treatment, and post fertilization. These depictions summarize the localization patterns of LACTB, PRDX3, PSMA8, TOMM34, and FUNDC1. SC—spermatocyte; RS—round spermatid; ES—elongating spermatid; Sp—spermatozoon, PrS—primed spermatozoon; CFS1—primed spermatozoon treated with MII oocyte extract for 4 h; CFS2—primed spermatozoon treated with MII oocyte extract for 24 h; and ZYG—zygote.

In this study, five candidate mitophagy proteins of interest were studied by utilizing the porcine CFS as well as porcine IVF in conjunction with immunocytochemistry (ICC) to compare protein localization patterns within each system at different time points. Protein distribution patterns in spermatids, spermatozoa (control and CFS treated) and zygotes are summarized in Fig. 8**.** These trials were conducted to characterize the target proteins’ dynamics during early fertilization, focusing on exploring their potential roles in post-fertilization mitophagy. Four out of five examined proteins have been associated with the cellular processes of mitophagy (FUNDC1) and/or mitochondrial homeostasis (LACTB, PRDX3, TOMM34). Furthermore, protein interactome analysis revealed multiple associations of the examined proteins with proteins previously implicated in post-fertilization sperm mitophagy, including SQSTM1, GABARP and LC3A [5]. To our knowledge, proteasomal subunit PSMA8 is not directly associated with autophagy or mitochondria but could be linked to post-fertilization mitophagy via VCP, a translocase serving ubiquitinated proteins to 26S proteasome [8]. Our observations of Class 2 protein PSMA8 and Class 3 protein TOMM34 matched their mass spectrometry classifications when evaluated by ICC. However, we observed that in reference to the Class 1 proteins, some of the findings did not completely reflect the mass spectrometry classification. According to the mass spectrometry results, LACTB was not detectable in primed spermatozoa, but when we examined LACTB by using ICC, we observed that a weak LACTB labeling was detected along the tails of primed spermatozoa. This could be a result of LACTB protein unmasking, but also non-specific antibody binding caused by sperm demembranation or disulfide bond removal. Regarding the ejaculated, non-capacitated 4- and 24-h CFS-exposed spermatozoa, our observations of LACTB agree with the mass spectrometry results. PRDX3 is also a Class 1 protein whose observations did not completely align with the mass spectrometry results. According to the mass spectrometry results, PRDX3 was not found in the ejaculated, non-capacitated or primed spermatozoa. However, we detected PRDX3 by ICC/epifluorescence microscopy in the connecting piece of the ejaculated, non-capacitated spermatozoa as well as in the primed spermatozoa with the addition of PRDX3 localization along the tail. In the 4- and 24-h CFS treated spermatozoa, our observations did agree with the mass spectrometry results.

Lactamase beta is a mammalian active-site serine protein hydrolase that has evolved from a bacterial penicillin-binding protein. LACTB has been shown to perform essential functions in the synthesis and maintenance of peptidoglycan, protecting bacterial cells from outside stressors such as antibiotics. LACTB has also been found in mitochondria, and it is suggested to be directly or indirectly regulating mitochondrial phospholipid metabolism [18, 19]. It has been found that LACTB is localized to the intermembrane space of the mitochondria. Through polymerization**,** it forms long, stable filaments to create intramitochondrial membrane organization and micro-compartments within the mitochondria, therefore aiding in mitochondrial structure [19]. LACTB filaments can access length scales larger than the size of mitochondrial membrane compartments. These filaments can form structural and functional bridges between the inner and outer mitochondrial membrane. This suggests that LACTB may have further roles in mitochondrial morphology as well as in processes where strong membrane association is needed [20]). The exact function of LACTB remains unknown; however, it has been suggested that LACTB plays roles in regulating metabolic pathways, autophagy, and PIK3R3 expression. The PI3K/Akt/mTOR signaling pathway is an important mechanism in the regulation of autophagy. It has been found that overexpression of LACTB in human cells caused a decrease in the expression of PIK3R3 (phosphoinositide-3-kinase regulatory subunit 3), which is a regulatory subunit of the PI3K complex. This causes the partial inhibition/downregulation of the PI3K/AKT/mTOR signaling pathway and thereby promoting autophagy. Upregulation of LACTB also increases the expression of the key pro-autophagic protein LC3 which results in the increase of autophagosome formation, again indicating that LACTB has the potential to promote autophagy [21]. These findings have aided in cancer research by suggesting that upregulation of LACTB can prevent cell proliferation through autophagy-mediated apoptosis [22]. In the present study, utilizing the porcine CSF, we observed that the localization of LACTB did not completely agree with the mass spectrometry findings. According to the mass spectrometry results, LACTB was not detected in primed spermatozoa. However, when we examined LACTB by using ICC, we observed that LACTB is present along the tail of primed spermatozoa. In other regards, our findings are consistent with those of the mass spectrometry analysis. When analyzing the Western blots as well as the immunofluorescence of ejaculated, non-capacitated and 4-h CFS exposed spermatozoa, LACTB protein band or labeling was not detectable but reappeared in spermatozoa exposed to the 24-h CFS. This moonlighting labeling was consistent with the labeling observed in the zygotes at 5-, 15- and 25-h post-insemination. As mentioned above, LACTB is localized to the intermembrane space of the mitochondria, but the LACTB filaments have the ability to access length scales larger than the size of mitochondrial membrane compartments [20]. Suggesting that the observed LACTB labeling along the tail could be due to the filaments being able to span beyond the mitochondrial intermembrane space. So, the observed labeling of LACTB along the tail of the spermatozoa after priming could potentially be due to the removal of the sperm plasma membrane as well as disulfide bonds allowing for the ectopic localization of LACTB outside of the mitochondrial sheath. LACTB protein was also detected in the cytoplasm of spermatocytes. In the round spermatids, the protein signal remained in the developing head and cytoplasm, and during spermatid elongation, it localized to the outer acrosomal membrane, manchette, and cytoplasmic lobe. LACTB was not observed in the 4-h CFS exposed spermatozoa, but it was observed in the 24-h CFS exposed spermatozoa. This could mean that LACTB activity is not necessary until the final stages of post-fertilization mitophagy in zygotes, mimicked by 24-h sperm exposure to the CFS. LACTB may be promoting autophagic activity by potentially increasing LC3 activity or regulating PIK3R3 expression [21], therefore playing a role in post-fertilization mitophagy. To our knowledge, LACTB protein has not been previously detected in mammalian oocytes.

Peroxiredoxin 3 is an antioxidant enzyme that protects cells from oxidative stress, plays a role in signaling mediated by reactive oxygen species (ROS), and promotes cell survival. Spermatozoa have been shown to express six PRDX species, which are known to be critical for fertility [23]. PRDXs have been shown to maintain sperm viability and fertilizing competence, as well as maintain ROS homeostasis. Although ROS production is necessary for sperm capacitation, due to their regulatory role in the phosphorylation of key proteins, their levels must be tightly controlled to prevent damaging oxidative stress and to maintain sperm structural integrity [23]. It has been demonstrated that PRDXs are expressed throughout porcine spermatozoa before and after capacitation [24] as well as throughout the entirety of mouse spermatozoa [25]. Lack of PRDXs results in the damage of membranes through lipid peroxidation, elevated ROS levels leading to sperm plasma membrane disruption, DNA damage, mitochondrial dysregulation, and prohibition of sperm capacitation, as well as impaired ATP generation by mitochondria [25]. Studies have also shown PRDXs to be localized in human oocytes and assigned the role of mediating ROS production. PRDX3 was found to increase throughout oogenesis, showing the lowest levels in primary oocytes to the highest measured level in metaphase II oocytes [26]. In this study, we are interested in the role of PRDX3 specifically in post-fertilization sperm mitophagy. In our mass spectrometry results, PRDX3 was not identified in ejaculated, non-capacitated or primed boar spermatozoa. When detecting PRDX3 in ejaculated, non-capacitated spermatozoa and by means of Western blotting and ICC, the PRDX3 protein band was detected weakly, and the fluorescently labeled protein was localized to the sperm tail connecting piece. Although this labeling does not agree with our mass spectrometry results, if PRDX3 is playing a role in mediating ROS homeostasis, it would make sense for it to be present in ejaculated, non-capacitated spermatozoa as ROS are present. The PRDX family proteins have also been labeled throughout the spermatozoa length in epididymal, non-capacitated mouse spermatozoa [25] as well as in ejaculated, non-capacitated boar spermatozoa [24]. It is possible that because PRDX3 labeling is confined to such a small part of the spermatozoa, which may also be resistant to our mild protein extraction protocol, the mass spectrometry analysis found the amount of this protein to be below the detection threshold. After 4 and 24 h of exposure to our CFS, PRDX3 was still found to be localized to the connecting piece as well as throughout the tail of the spermatozoa, with increased labeling after the 24-h treatment. This labeling could be due to a passive release of ROS by dying spermatozoa during extract co-incubation [27]. This leads to an increase of PRDX3 which may be acting to regulate ROS production in order to prevent damaging oxidative stress and preserve sperm integrity [23]. This pattern of labeling was not found in the fertilizing spermatozoa of the 5-,15-, or 25-h post-insemination zygotes. The PRDX3 protein was found in the cytoplasm of the zygotes which is consistent with the PRDX3 localization found in human oocytes [26]. Furthermore, six PRDX family protein coding transcripts including PRDX3 transcript have been detected in bovine oocytes and embryos and proposed to participate in oocyte maturation and embryo development [28]. Likewise, PRDX3 could modulate ROS production in the porcine zygote.

Spermatogenesis is a highly ordered physiological process in which haploid male germ cells are produced through multiple steps and morphological and cellular transformations. Throughout spermatogenesis, dynamics such as an increase in the amount of 20S proteasomes in spermatids, replacement of PSMA7 by PSMA8 in spermatocytes, as well as assembly of PA200-20S proteasomes in spermatocytes and spermatids have been observed [17]. One of the known components that plays a role in the progression of spermatogenesis is proteasomal subunit alpha 8 (PSMA8). PSMA8 is a testis specific proteasomal subunit found during spermatogenesis that appears to be crucial for proper meiotic exit by the forming spermatids [29]. Consequently, there is no evidence of its expression in the female germ cells. Meiosis is a fundamental process in sexually reproducing species in which two rounds of cell division result in four cells with only one copy of each chromosome, ensuring the generation of genetically diverse haploid gametes [17]. Meiotic recombination takes place on the synaptonemal complex (SC), a protein structure that forms between homologous chromosomes, during prophase I. It has been demonstrated that PSMA8 is localized to the synaptonemal complex and promotes the assembly of the proteasome activator PA200 [17]. Proteasomes capped with PA200 are responsible for the degradation of acetylated histones to promote histone-to-protamine replacement during spermiogenesis. PSMA8 deficiency causes a significant decrease in PA200 level resulting in accumulation of the acetylated histones as well as a significant decrease in 20S catalytic subunits which ultimately results in abortive proteasome assembly [17]. Xiong et al*.* proposed that PA200-20S is the main proteasome isoform functioning in protein degradation in spermatocytes and spermatids [29]. It has also been shown that PSMA8 deficient mice show delayed spermatocyte entry into metaphase I and are arrested at this stage, resulting in few spermatids and mature spermatozoa in the seminiferous tubules or epididymis, and overall, male infertility [17, 29]. When observing PSMA8 in ejaculated, non-capacitated spermatozoa in both the Western blot and immunofluorescence of primed spermatozoa, it was detectable as a protein band of predicted size and localized to the acrosome. This localization pattern is expected, as the sperm acrosome contains an abundance of 26S proteasomes and consequently the various proteasomal subunits [30]. Moreover, in elongating spermatids, PSMA8 localized to the acrosomal and post-acrosomal regions, with increased signal intensity observed along the outer edge of the acrosome. There could also be some carryover of synaptonemal protein residues and fragments in the spermatozoa head. This acrosomal localization pattern can be observed in ejaculated, non-capacitated spermatozoa for other proteasomal subunits, including subunit PSMA3 and proteasome assembly protein PSMG2 [30]. Notably, PSMG2 is involved in the regulation of proteasome-autophagy balance [31] and localizes to the sperm acrosome in primed and CFS-exposed spermatozoa [10]. After 4- and 24- hours of exposure to CFS, the PSMA8 localization pattern shifts from the head of the spermatozoa to the connecting piece and principal piece of the tail. Sperm priming for CFS may remove most of the proteasomes residing in the outer acrosomal membrane and acrosomal matrix and only leave the proteasomal subunits of the extraction-resistant inner acrosomal membrane intact [32]. This suggests that the proteasome assembly pathways may remain active in the cell-free system. Likely, a combination of sperm-borne and ooplasm-derived proteasomal subunits and regulators are observed and interacting within the CFS. The ubiquitin–proteasome system has already been implicated in post-fertilization sperm mitophagy [1]. As observed in the mass spectrometry results, the increase of these subunits and regulators is to be expected. When observing PSMA8 localization 5 h post-fertilization, it was detected in the zygotic cytoplasm as well as in the connecting piece and along the entire tail of the fertilizing spermatozoa. The ubiquitin proteasome system is known to assist with sperm mitochondrion degradation after fertilization, so this localization pattern within zygotes is expected. Early in fertilization, proteasomes have previously been described in the connecting piece of human and bovine spermatozoa [33]. It has been suggested that the proteasomes assist in the disassembly of connecting piece structures, exposing the sperm centriole to the zygotic cytoplasm which is an essential step in the formation of an active zygotic centrosome [33]. In zygotes at 15 h post-insemination, PSMA8 was detected abundantly in the cytoplasm and appeared to be associated with forming PPN; this localization pattern is expected, as the proteasomes are known to assist with PN development, including but not limited to sperm protamine removal [17]. In zygotes 25 h post-insemination, PSMA8 was still detected in the cytoplasm, but it no longer appeared to be associated with the pronuclei. Perhaps in the CFS, we are observing some proteasomal activity in the connecting piece and tail of the spermatozoa, based on the localization of PSMA8.

Translocase of outer mitochondrial membrane 34 (TOMM34) is a chaperone-like protein which imports precursor proteins into the mitochondria. TOMM34 has been found within the cell cytoplasm as well as associated with the outer mitochondrial membrane. Most mitochondrial membrane proteins are synthesized in the cytoplasm and must be imported into the mitochondria in an unfolded form. Cytosolic chaperones with ATPase activity such as HSP70 and HSP90 are part of a large cytosolic complex that delivers proteins to the mitochondrion in mammalian cells. TOMM34 has been found to assemble into the cytosolic complex as a specific cochaperone of HSP70/HSP90 for mitochondrial protein import. Mammalian cells utilize both HSP90 and HSP70 for the transfer of preproteins to the translocase of the outer mitochondria membrane (TOM complex) post-translationally. As a cochaperone of HSP90/HSP70, TOMM34 could regulate the ATPase activity of the HSP chaperones and influence the substrate binding [34]. In previous studies, TOMM34 has been detected in the sperm head as well as in the mitochondria [35, 36]. The localization of TOMM34 in the head of spermatozoa has also been observed in the present study, in addition to whole tail labeling in control spermatozoa. However, after 24 h of sperm-CFS exposure, TOMM34 localization was confined to the mitochondrial sheath. These findings agree with the mass spectrometry findings as well as the Class 3 categorization given to the TOMM34 protein in our proteomic study. Mirroring the 24-h CFS pattern, clustering of TOMM34 on the fertilizing spermatozoa’s mitochondrial sheath observing the zygotes 15- and 25- hour post-insemination. The reduction in TOMM34 labeling from ooplasm-exposed spermatozoa may be related to a role in destabilizing sperm mitochondrial membrane and prepping the mitochondrial sheath for degradation. The presence of TOMM34 in oocyte cytoplasm is corroborated by the identification of its transcript in the human, Rhesus macaque, bovine and murine oocytes [37].

Mammalian mitophagy receptor FUNDC1 (FUN14 domain containing 1) is expressed on the mitochondrial outer membrane and contributes to mitochondrial quality control following hypoxic stress. Although the factors that initiate and mediate the degradation of paternal mitochondria following fertilization remain unknown, FUNDC1 was identified based on its ability to facilitate the depletion of mitochondria damaged by exposure to hypoxia and its selective association with pro-autophagic microtubule-associated proteins 1A/1B light chain 3B (MAP1LC3B/LC3) [38]. It has been found that mitophagy involves mitochondrial outer membrane receptors that can recruit autophagosomes through direct binding to LC3, such as FUNDC1 [39]. Mitochondria are highly dynamic organelles that undergo constant fusion and fission. FUNDC1 has also been found to play a role in the coordination of the signaling pathway that is mediating this fusion and fission [40] implying that FUNDC1 plays a role in mitochondrial quality control by regulating mitochondrial biogenesis and mitophagy. It has been suggested that FUNDC1 also mediates a “coupling” mechanism between mitochondrial dynamics and receptor-mediated mitophagy at molecular level in mammalian cells. The mitochondrial fission factor DNM1L, and the mitochondrial inner membrane fission or fusion protein OPA1, both interact with FUNDC1 for receptor-induced mitophagy [41]. Although FUNDC1 plays a plethora of roles in the mitochondria, it was not identified in our mass spectrometry proteomics results. However, its homolog FUNDC2, localized to the outer membrane of the mitochondria and essential for serine/threonine kinase AKT1 signaling, was identified as a Class 3 protein in our proteomic CFS study [10]. Nevertheless, FUNDC1 was detected in ejaculated, non-capacitated spermatozoa via Western blotting as well as lightly in the mitochondrial sheath of ejaculated, non-capacitated spermatozoa by using immunocytochemistry. This faint mitochondrial signal could be due to disulfide bonds limiting sperm mitochondrial exposure to FUNDC1 antibodies. After sperm priming, FUNDC1 was detected on the head of the spermatozoa as well as throughout the sperm tail with a brightest signal in the mitochondrial sheath, possibly due to the removal of disulfide bonds by DTT. This signal persisted after 4 h of exposure to CFS, while at 24 h of CFS, FUNDC1 was confined to the mitochondrial sheath. In the 5-h post-insemination zygotes, FUNDC1 was detected in the zygotic cytoplasm as well as on the acrosome of the fertilizing spermatozoa. At 15 h post-insemination, FUNDC1 was found in the cytoplasm of the zygotes as well as associated with the paternal pronuclei, and possibly assisting in mitochondrial sheath degradation. The presence of FUNDC1 in the early stage paternal pronuclei could be a result of its dissipation from the solubilizing sperm head perinuclear theca or the decondensing sperm nucleus, in agreement with our observation of FUNDC1 in the sperm head and spermatid nuclei. Alternatively, FUNDC1 and other autophagy-associated proteins of ooplasmic origin could be sequestered in the (pro)nuclear space in the form of membraneless biomolecular condensates (MBCs; [42]). These small multi-protein aggregates have been implicated in the varied but interconnected cellular processes, including autophagy [43]. The association of FUNDC1 with the zygotic paternal pronuclei and the mitochondrial sheaths of the fertilizing spermatozoa was lost by 25-h post-insemination. In agreement with our findings, FUNDC1 has been found recently in *C. elegans* oocytes and zygotes and deemed required for maintenance mitophagy during oocyte-to-zygote transition [44].

The common denominator of the developmental origin of target proteins discussed above appears to be their diffuse labeling in spermatocytes, hinting at their synthesis during meiosis, which is common for proteins involved in spermatid development [45]. Throughout the haploid phase, diffuse or granular localization in the cytoplasm, now becoming a polarized cytoplasmic lobe, carries over from meiotic phase. Concentrated localization patterns emerge post-meiotically, including association with the developing acrosome and/or mitochondrial sheath, generally agreeing with final localization of individual target proteins in the ejaculated spermatozoa.

In conclusion, findings from our previous mass spectrometry trial informed the present study and enabled us to validate five candidate sperm mitophagy determinants by characterizing their localization patterns in various spermatogenic cell types, ejaculated, non-capacitated spermatozoa, IVF zygotes, and primed spermatozoa exposed to our unique CFS. Functional studies, such as how inhibition of these specific proteins’ activities would affect post-fertilization sperm mitochondrial degradation, will be conducted to further define their role in this process. Nevertheless, the results of this phenotyping study already show that our mass spectrometry-based mitophagy protein classification data can be replicated in the porcine CSF and in the zygote. It should be noted that some of our proteins deviated in their localization within zygotes when compared to the CFS. As well as some proteins’ localization in the CFS did not completely agree with what was expected based on the mass spectrometry results. These deviations may be caused by a dilution or unintentional removal of some ooplasmic factors within the CFS or perhaps the CFS lacking in concentration of some factors which allow for more robust protein localization patterns seen in the zygotes. These differences highlight the importance of using in vitro fertilization to validate CFS and proteomic findings. However, the inventory of autophagic/mitophagic proteins which lead to the identification of candidate determinants of mitochondrial inheritance was only made possible by our cell-free system.

Altogether, present results validate our porcine CFS and advance the understanding of mitochondrial inheritance, potentially shedding light on the origins of certain mitochondrial diseases arising from the failure of paternal mitochondrial genome elimination at fertilization. This novel system will thus remain a useful tool for a molecular level exploration of early post-fertilization events guiding the processing of accessory sperm structures such as mitochondrial sheath, fibrous sheath and axoneme, but also studies of pronuclear development, zygotic genome activation, and centrosomal inheritance [46]. Future studies will define the function of these pro-autophagic proteins by using gene ablation, loss of function, and pharmacological inhibition to alter post-fertilization sperm mitochondrial degradation.

## Supplementary Information

Below is the link to the electronic supplementary material.

Supplementary Material 1

## Data Availability

Upon request.

## References

[1] Sutovsky P, Moreno RD, Ramalho-Santos J, Dominko T, Simerly C, Schatten G. Ubiquitin tag for sperm mitochondria. Nature. 1999;402(6760):371–2. 10586873 10.1038/46466

[2] Sutovsky P, Navara CS, Schatten G. Fate of the sperm mitochondria, and the incorporation, conversion, and disassembly of the sperm tail structures during bovine fertilization. Biol Reprod. 1996;55(6):1195–205. 8949874 10.1095/biolreprod55.6.1195

[3] Sutovsky P, Moreno RD, Ramalho-Santos J, Dominko T, Simerly C, Schatten G. Ubiquitinated sperm mitochondria, selective proteolysis, and the regulation of mitochondrial inheritance in mammalian embryos. Biol Reprod. 2000;63(2):582–90. 10906068 10.1095/biolreprod63.2.582

[4] Sutovsky P, McCauley TC, Sutovsky M, Day BN. Early degradation of paternal mitochondria in domestic pig (Sus scrofa) is prevented by selective proteasomal inhibitors lactacystin and MG132. Biol Reprod. 2003;68(5):1793–800. 12606393 10.1095/biolreprod.102.012799

[5] Song WH, Yi YJ, Sutovsky M, Meyers S, Sutovsky P. Autophagy and ubiquitin-proteasome system contribute to sperm mitophagy after mammalian fertilization. Proc Natl Acad Sci U S A. 2016;113(36):E5261–70. 27551072 10.1073/pnas.1605844113 PMC5018771

[6] Zuidema D, Sutovsky P. The domestic pig as a model for the study of mitochondrial inheritance. Cell Tissue Res. 2019. 10.1007/s00441-019-03100-z. 31511985

[7] Sutovsky P, Kerns K, Zigo M, Zuidema D. Boar semen improvement through sperm capacitation management, with emphasis on zinc ion homeostasis. Theriogenology. 2019;137:50–5. 31235187 10.1016/j.theriogenology.2019.05.037

[8] Song W-H, Zuidema D, Yi Y-J, Zigo M, Zhang Z, Sutovsky M, et al. Mammalian cell-free system recapitulates the early events of post-fertilization sperm mitophagy. Cells. 2021;10(9):2450. 34572103 10.3390/cells10092450 PMC8466530

[9] Song WH and Sutovsky P. Porcine cell-free system to study mammalian sperm mitophagy. Methods Mol Biol, 2018 10.1007/7651_2018_158 29749586

[10] Zuidema D, Jones A, Song WH, Zigo M, Sutovsky P. Identification of candidate mitochondrial inheritance determinants using the mammalian cell-free system. Elife. 2023. 10.7554/eLife.85596.3. 37470242 PMC10393022

[11] Zigo M, Kerns K, Sutovsky P. Methods of mammalian in vitro sperm capacitation to study sperm physiology. Methods Mol Biol. 2025;2954:229–40. 40601279 10.1007/978-1-0716-4698-4_13

[12] Sutovsky P. Visualization of sperm accessory structures in the mammalian spermatids, spermatozoa, and zygotes by immunofluorescence, confocal, and immunoelectron microscopy. Methods Mol Biol. 2004;253:59–77. 15037788 10.1385/1-59259-744-0:059

[13] Chen PR, Redel BK, Spate LD, Ji T, Salazar SR, Prather RS. Glutamine supplementation enhances development of in vitro-produced porcine embryos and increases leucine consumption from the medium. Biol Reprod. 2018;99(5):938–48. 29860318 10.1093/biolre/ioy129 PMC6297286

[14] Sutovsky P, Tengowski MW, Navara CS, Zoran SS, Schatten G. Mitochondrial sheath movement and detachment in mammalian, but not nonmammalian, sperm induced by disulfide bond reduction. Mol Reprod Dev. 1997;47(1):79–86. 9110318 10.1002/(SICI)1098-2795(199705)47:1<79::AID-MRD11>3.0.CO;2-V

[15] Chidlow G, Wood JP, Knoops B, Casson RJ. Expression and distribution of peroxiredoxins in the retina and optic nerve. Brain Struct Funct. 2016;221(8):3903–25. 26501408 10.1007/s00429-015-1135-3 PMC5065902

[16] Pirson M, Clippe A, Knoops B. The curious case of peroxiredoxin-5: what its absence in aves can tell us and how it can be used. BMC Evol Biol. 2018;18(1):18. 29422028 10.1186/s12862-018-1135-z PMC5806436

[17] Gómez HL, Felipe-Medina N, Condezo YB, Garcia-Valiente R, Ramos I, Suja JA, et al. The PSMA8 subunit of the spermatoproteasome is essential for proper meiotic exit and mouse fertility. PLoS Genet. 2019;15(8):e1008316. 31437213 10.1371/journal.pgen.1008316 PMC6726247

[18] Peitsaro N, Polianskyte Z, Tuimala J, Pörn-Ares I, Liobikas J, Speer O, et al. Evolution of a family of metazoan active-site-serine enzymes from penicillin-binding proteins: a novel facet of the bacterial legacy. BMC Evol Biol. 2008;8:26. 18226203 10.1186/1471-2148-8-26 PMC2266909

[19] Polianskyte Z, Peitsaro N, Dapkunas A, Liobikas J, Soliymani R, Lalowski M, et al. LACTB is a filament-forming protein localized in mitochondria. Proc Natl Acad Sci U S A. 2009;106(45):18960–5. 19858488 10.1073/pnas.0906734106 PMC2767363

[20] Bennett JA, Steward LR, Rudolph J, Voss AP, Aydin H. The structure of the human LACTB filament reveals the mechanisms of assembly and membrane binding. PLoS Biol. 2022;20(12):e3001899. 36534696 10.1371/journal.pbio.3001899 PMC9815587

[21] Xu W, Yu M, Qin J, Luo Y, Zhong M. LACTB regulates PIK3R3 to promote autophagy and inhibit EMT and proliferation through the PI3K/AKT/mTOR signaling pathway in colorectal cancer. Cancer Manag Res. 2020;12:5181–200. 32636680 10.2147/CMAR.S250661 PMC7335311

[22] Yang F, Yan Z, Nie W, Cheng X, Liu Z, Wang W, et al. LACTB induced apoptosis of oxaliplatin-resistant gastric cancer through regulating autophagy-mediated mitochondrial apoptosis pathway. Am J Transl Res. 2021;13(2):601–16. 33594312 PMC7868839

[23] O’Flaherty C, Boisvert A, Manku G, Culty M. Protective role of peroxiredoxins against reactive oxygen species in neonatal rat testicular gonocytes. Antioxidants (Basel). 2019;9(1):32. 31905831 10.3390/antiox9010032 PMC7022870

[24] Kwon WS, Rahman MS, Lee JS, Kim J, Yoon SJ, Park YJ, et al. A comprehensive proteomic approach to identifying capacitation related proteins in boar spermatozoa. BMC Genom. 2014;15(1):897. 10.1186/1471-2164-15-897 PMC4287242 25315394

[25] Ryu DY, Kim KU, Kwon WS, Rahman MS, Khatun A, Pang MG. Peroxiredoxin activity is a major landmark of male fertility. Sci Rep. 2017;7(1):17174. 29215052 10.1038/s41598-017-17488-7 PMC5719347

[26] Rodríguez-Nuevo A, Torres-Sanchez A, Duran JM, De Guirior C, Martínez-Zamora MA, Böke E. Oocytes maintain ROS-free mitochondrial metabolism by suppressing complex I. Nature. 2022;607(7920):756–61. 35859172 10.1038/s41586-022-04979-5 PMC9329100

[27] Gualtieri R, Kalthur G, Barbato V, Longobardi S, Di Rella F, Adiga SK, et al. Sperm oxidative stress during in vitro manipulation and its effects on sperm function and embryo development. Antioxidants. 2021. 10.3390/antiox10071025. 34202126 PMC8300781

[28] Leyens G, Knoops B, Donnay I. Expression of peroxiredoxins in bovine oocytes and embryos produced in vitro. Mol Reprod Dev. 2004;69(3):243–51. 15349835 10.1002/mrd.20145

[29] Xiong Y, Yu C, Zhang Q. Ubiquitin-proteasome system-regulated protein degradation in spermatogenesis. Cells. 2022. 10.3390/cells11061058. 35326509 PMC8947704

[30] Sutovsky P. Sperm proteasome and fertilization. Reproduction. 2011;142(1):1–14. 21606061 10.1530/REP-11-0041

[31] Wang X, Yu J, Liu X, Luo D, Li Y, Song L, et al. PSMG2-controlled proteasome-autophagy balance mediates the tolerance for MEK-targeted therapy in triple-negative breast cancer. Cell Rep Med. 2022;3(9):100741. 36099919 10.1016/j.xcrm.2022.100741 PMC9512673

[32] Yi Y-J, Manandhar G, Sutovsky M, Zimmerman SW, Jonáková V, Van Leeuwen FW, et al. Interference with the 19S proteasomal regulatory complex subunit PSMD4 on the sperm surface inhibits sperm-zona pellucida penetration during porcine fertilization. Cell Tissue Res. 2010;341(2):325–40. 20526895 10.1007/s00441-010-0988-2

[33] Rawe VY, Díaz ES, Abdelmassih R, Wójcik C, Morales P, Sutovsky P, et al. The role of sperm proteasomes during sperm aster formation and early zygote development: implications for fertilization failure in humans. Hum Reprod. 2008;23(3):573–80. 18089554 10.1093/humrep/dem385

[34] Faou P, Hoogenraad NJ. Tom34: a cytosolic cochaperone of the Hsp90/Hsp70 protein complex involved in mitochondrial protein import. Biochim Biophys Acta. 2012;1823(2):348–57. 22178133 10.1016/j.bbamcr.2011.12.001

[35] Chewawiwat N, Yano M, Terada K, Hoogenraad NJ, Mori M. Characterization of the novel mitochondrial protein import component, Tom34, in mammalian cells. J Biochem. 1999;125(4):721–7. 10101285 10.1093/oxfordjournals.jbchem.a022342

[36] Zhang M, Chiozzi RZ, Skerrett-Byrne DA, Veenendaal T, Klumperman J, Heck AJR, et al. High resolution proteomic analysis of subcellular fractionated boar spermatozoa provides comprehensive insights into perinuclear theca-residing proteins. Front Cell Dev Biol. 2022;10:836208. 35252197 10.3389/fcell.2022.836208 PMC8894813

[37] Biase FH. Oocyte developmental competence: insights from cross-species differential gene expression and human oocyte-specific functional gene networks. OMICS. 2017;21(3):156–68. 28253086 10.1089/omi.2016.0177

[38] Wu W, Lin C, Wu K, Jiang L, Wang X, Li W, et al. FUNDC1 regulates mitochondrial dynamics at the ER-mitochondrial contact site under hypoxic conditions. EMBO J. 2016;35(13):1368–84. 27145933 10.15252/embj.201593102 PMC4864280

[39] Yoo SM, Jung YK. A molecular approach to mitophagy and mitochondrial dynamics. Mol Cells. 2018;41(1):18–26. 29370689 10.14348/molcells.2018.2277 PMC5792708

[40] Lim Y, Rubio-Peña K, Sobraske PJ, Molina PA, Brookes PS, Galy V, et al. Fndc-1 contributes to paternal mitochondria elimination in C. elegans. Dev Biol. 2019;454(1):15–20. 31233739 10.1016/j.ydbio.2019.06.016 PMC6717525

[41] Chen M, Chen Z, Wang Y, Tan Z, Zhu C, Li Y, et al. Mitophagy receptor FUNDC1 regulates mitochondrial dynamics and mitophagy. Autophagy. 2016;12(4):689–702. 27050458 10.1080/15548627.2016.1151580 PMC4836026

[42] Schmit JD, Feric M, Dundr M. How hierarchical interactions make membraneless organelles tick like clockwork. Trends Biochem Sci. 2021;46(7):525–34. 33483232 10.1016/j.tibs.2020.12.011 PMC8195823

[43] Li R, Li Y, Wu S, Zhou Z, Hou X, Yang P, et al. Proximal proteomics reveals a landscape of human nuclear condensates. Nat Cell Biol. 2025;27(12):2198–213. 41315769 10.1038/s41556-025-01809-4

[44] Thendral SB, Bacot S, Ryde IT, Morton KS, Chi Q, Kenny-Ganzert IW, et al. Programmed mitophagy at the oocyte-to-zygote transition promotes lineage endurance. Nat Cell Biol. 2026;28(2):268–84. 41526527 10.1038/s41556-025-01854-z PMC12918445

[45] Barth A, Perry VEA, Hamilton LE, Sutovsky P, Oko R. Bovine spermatogenesis. Adv Anat Embryol Cell Biol. 2025;240:65–136. 40272587 10.1007/978-3-031-70126-9_2

[46] Sutovsky P, Zigo M, Tirpak F, Oko R. Paternal contributions to mammalian zygote - beyond sperm-oocyte fusion. Curr Top Dev Biol. 2025;162:387–446. 40180516 10.1016/bs.ctdb.2025.02.002

